# H3K18 lactylation-mediated SPHK1-SIRT1 feedback loop accelerates pyroptosis of tubular epithelial cells in sepsis-associated acute kidney injury

**DOI:** 10.7150/thno.122991

**Published:** 2026-02-18

**Authors:** Yan Huang, Eryang Zhao, Guangyu Zhao, Wenfeng Zhuo, Yingsong Zhao, Hongda Wang, Guozheng Lv, Rong Hu, Zhu Zeng, Shengbo Han, Yuhang Hu, Gang Zhao

**Affiliations:** Department of Emergency Surgery, Union Hospital, Tongji Medical College, Huazhong University of Science and Technology, Wuhan, China.

**Keywords:** H3K18 lactylation, SA-AKI, SPHK1, SIRT1, pyroptosis

## Abstract

**Background:**

Lactate accumulation exacerbates the severity of sepsis-associated acute kidney injury (SA-AKI), although the mechanism remains unclear. Since pyroptosis contributes to renal tubular epithelial cell (RTEC) death during SA-AKI, this study explores whether lactate exacerbates pathogenesis by promoting RTEC pyroptosis.

**Methods:**

The clinical correlation between lactate and SA-AKI was examined using the Medical Information Mart for Intensive Care IV (MIMIC-IV) database and patient samples. Lactate's role in RTEC pyroptosis was evaluated in lipopolysaccharide (LPS)-exposed HK-2 cells and in cecal ligation and puncture (CLP)-induced mice. Cross-analyzing bioinformatics and RNA-seq data from LPS/lactate-exposed HK-2 cells revealed pyroptosis genes associated with SA-AKI. Molecular mechanisms were explored via Western blot, ELISA, mitochondrial function assays, chromatin immunoprecipitation (ChIP), and co-immunoprecipitation (co-IP). High-throughput drugs screening was conducted to identify candidates acting on the Sphingosine kinase 1(SPHK1)/Sirtuin 1(SIRT1) axis, which were validated *in vitro* and *in vivo*.

**Results:**

Lactate aggravated SA-AKI by promoting RTEC pyroptosis. Bioinformatic and functional studies identified SPHK1 as the key mediator. Both SPHK1 knockdown and its inhibitor PF-543 alleviated lactate-augmented pyroptosis. Drug screening identified nicotinamide adenine dinucleotide (NAD^+^), which simultaneously suppressed SPHK1 expression and the RTEC injury marker kidney injury molecule-1 (KIM-1). Combining NAD^+^ and PF-543 synergistically attenuated SA-AKI. Sepsis-induced lactate accumulation promoted P300-mediated histone H3 lysine 18 lactylation (H3K18la) at the SPHK1 promoter, epigenetically enhancing its transcription. SPHK1 then phosphorylated and degraded SIRT1, inducing peroxisome proliferator-activated receptor gamma co-activator 1α (PGC-1α) hyperacetylation, thereby impairing SIRT1/PGC-1α signaling and triggering NOD-like receptor family pyrin domain containing 3 (NLRP3) inflammasome-driven pyroptosis. Reciprocally, SIRT1 acted as a delactylase delactylase to reduce H3K18la and inhibit SPHK1 transcription, forming a SPHK1-SIRT1 negative feedback loop.

**Conclusions:**

The study identifies an H3K18la-mediated SPHK1-SIRT1 axis as a key factor of RTEC pyroptosis in SA-AKI. The combined pharmacological strategy of NAD^+^ supplementation and SPHK1 inhibition represents a promising therapeutic strategy for SA-AKI.

## Introduction

Sepsis-associated acute kidney injury (SA-AKI) is a widespread and life-threatening complication in critically ill patients, with significantly high mortality rates 1,2. This poor prognosis stems from a complex pathophysiology involving innate immune activation, endothelial dysfunction, mitochondrial damage, and tubular epithelial injury 3, for which current treatment strategies remain mainly supportive and non-targeted (e.g., fluid resuscitation and renal replacement therapy) 4,5. Consequently, there is a pressing need for therapies that directly intercept core injury mechanisms. Recent evidence has demonstrated that pyroptosis, a lytic inflammatory type of programmed cell death caused by gasdermin pore formation, plays a crucial role in kidney tubular injury in SA-AKI 6,7. This caspase-dependent process is triggered by NLRP3 inflammasome activation in response to mitochondrial damage and oxidative stress, which not only eliminates infected or damaged cells but also amplifies systemic inflammation via Interleukin-1 beta (IL-1β)/Interleukin-18 (IL-18) release. Evidence also showed that caspase-11-mediated pyroptosis in RTEC was essential in SA-AKI pathogenesis 7. Importantly, targeted inhibition of the NLRP3/caspase-1/Gasdermin D(GSDMD) pyroptosis pathway has been shown to provide therapeutic benefits for SA-AKI 8, supporting its potential as a treatment strategy for sepsis-induced kidney injury.

Lactate, which is the end-product of glycolysis, serves as a biomarker and an independent prognostic indicator in sepsis, correlating with death and multi-organ dysfunction 9,10. Emerging evidence has identified hyperlactatemia as a factor that worsens kidney injury during sepsis 11,12. This effect is mediated through lactylation of histones and non-histones proteins, which promotes sepsis-associated organ injury by remodeling chromatin and activating inflammatory genes 13,14. Recent studies have demonstrated that accumulated lactate induces mitochondrial fission 1 protein (FIS1) lactylation and worsens SA-AKI by amplifying oxidative stress 11,15-17. Notably, emerging evidence has revealed lactylation-mediated pyroptosis pathways in different injury models. While histone lactylation activates caspase-1-dependent pyroptosis in cerebral ischemia-reperfusion injury 18, non-histone lactylation triggers caspase-11-mediated non-canonical pyroptosis in acetaminophen-induced liver toxicity 19. Despite these advances linking post-translational modifications to pyroptosis, whether lactylation influences pyroptosis in SA-AKI remains unknown.

Sphingosine kinase 1 (SPHK1), the enzyme responsible for producing bioactive sphingosine-1-phosphate 20, is a known regulator of inflammatory responses and contributes to sepsis pathophysiology 21. SPHK1 overexpression directly correlates with sepsis-induced multi-organ injury 21,22. In response to LPS, SPHK1 is upregulated across various cell types (e.g., macrophages, microglia, Kupffer cells and pulmonary endothelial cells), where it initiates a harmful feedback loop by amplifying pro-inflammatory cytokine secretion and disrupting immune homeostasis. While SPHK1's role in systemic inflammation and extra-renal sepsis complications through enhancing cytokine storms and disrupting immune homeostasis is established, its involvement in SA-AKI remains undefined. Thus, this study aims to determine whether SPHK1 serves as the critical link between septic metabolic stress and renal tubular injury.

This study demonstrates that lactate aggravates RTEC pyroptosis both *in vitro* and *in vivo*. Through an integrated analysis of bioinformatics and RNA-sequencing data, the study identified SPHK1 as a crucial regulator of lactate-induced pyroptosis in RTEC. The study further investigated whether SPHK1/SIRT1/PGC-1α-mediated mitochondrial homeostasis contributes to lactate-induced pyroptosis and elucidated the epigenetic mechanism by which lactate regulates SPHK1 expression. Additionally, drug screening was employed to explore compounds modulating SPHK1 lactylation and to evaluate their therapeutic potential in SA-AKI. These findings establish lactate-mediated RTEC pyroptosis as a therapeutic target to disrupt metabolic-epigenetic-inflammatory crosstalk in SA-AKI.

## Results

### Lactate exacerbates SA-AKI via pyroptosis-dependent tubular damage

**Figure 1:**
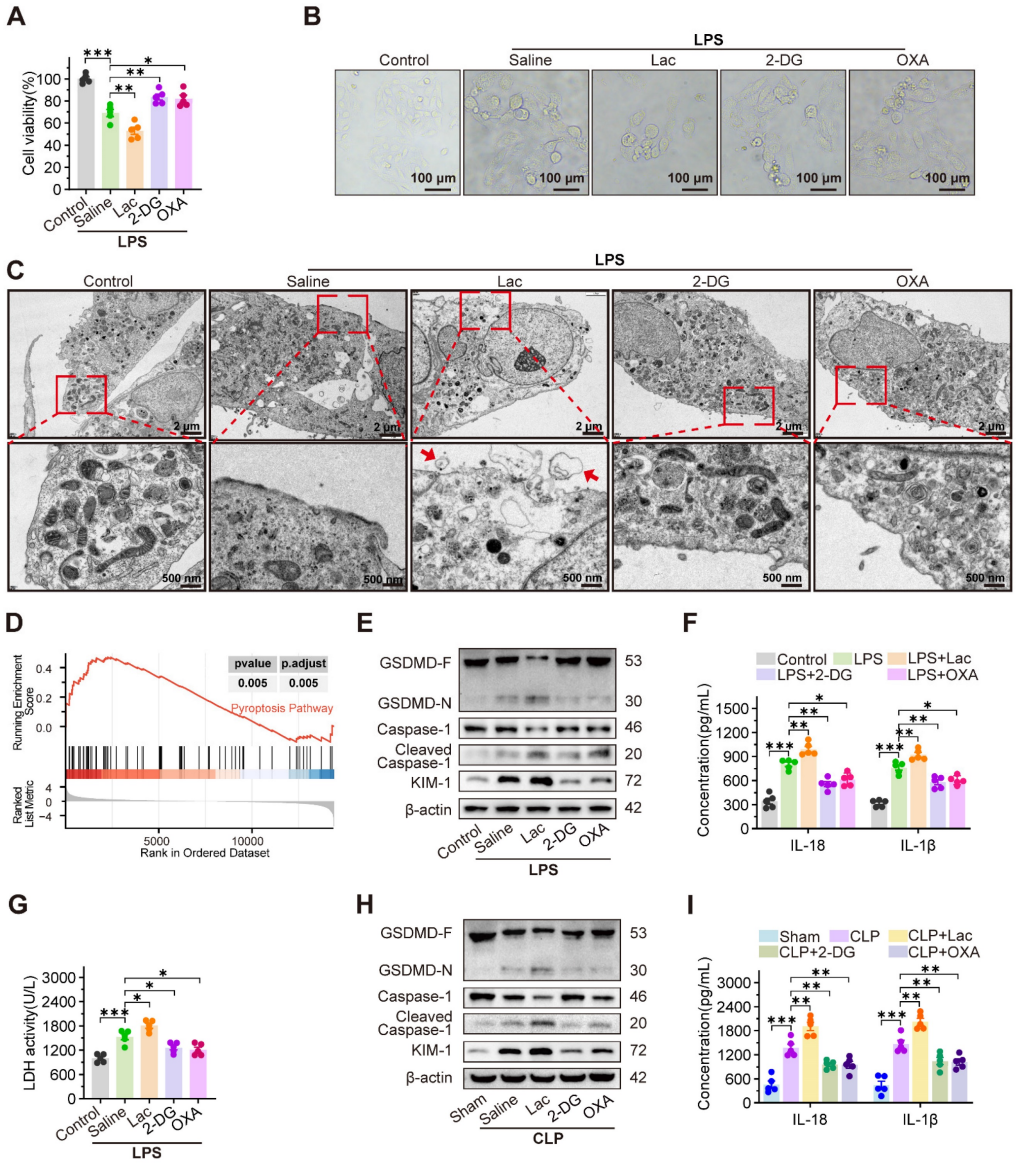
** Lactate exacerbates SA-AKI via pyroptosis-dependent tubular damage.** (A) Cell viability and (B) representative morphological features of pyroptosis (bright-field, scale bar = 100 μm), and (C) transmission electron microscopy (TEM) images. (Scale bars = 2 μm, up; 500 nm, down) in HK-2LPS cells treated with lactate/2-DG/oxamate (n = 5). (D) Enrichment plot of pyroptosis pathway via GSEA analysis. (E) Protein levels of GSDMD, Caspase-1, Cleaved Caspase-1, and KIM-1, (F) ELISA analysis of IL-18 and IL-1β levels and (G) LDH release in in HK-2LPS cells treated with lactate/2-DG/oxamate (n = 5). (H) Protein levels of GSDMD, Caspase-1, Cleaved Caspase-1, and KIM-1 and (I) ELISA analysis of IL-18 and IL-1β levels in kidney tissue of CLP mice treated with lactate/2-DG/oxamate (n = 5). Data are mean ± SEM. *p < 0.05, **p < 0.01, and ***p < 0.001; ns, not significant.

To investigate the cytotoxicity of lactate on RTEC, HK-2 cells were treated with LPS to establish an *in vitro* sepsis model. As expected, both extracellular and intracellular lactate levels of HK-2 cells were increased after treatment with LPS (Figure S1A-B). In CLP-induced SA-AKI mice, serum and kidney tissue lactate levels were also significantly elevated (Figure S1C-D). Considering the elevated lactate in SA-AKI, we further evaluated whether lactate itself was capable of damaging RTEC. However, the cell viability assay showed that low concentrations of lactate (lower than 10 mM) failed to induce cytotoxicity in HK-2 cells (Figure S1E). Consistently, the *in vivo* experiments also demonstrated that high-dose lactate (up to 20 mM) alone caused minor kidney damage (Figure S1F). These results indicated that lactate was not the sole damage factor for SA-AKI. Therefore, we further investigated whether lactate synergistically damaged RTEC with LPS during SA-AKI. As expected, LPS-induced cytotoxicity was intensified with 5 mM lactate supplementation, yet it was considerably mitigated by the glycolysis inhibitors 2-deoxy-D-glucose (2-DG) and oxamate (OXA) (Figure 1A). Meanwhile, HK-2 cells exposed to both LPS and lactate showed morphological features characteristic of pyroptosis, including cell swelling, membrane rupture, and cytoplasmic content release (Figure 1B).

Notably, transmission electron microscopy (TEM) images revealed morphological characteristics indicative of pyroptosis (Figure 1C). This morphological evidence was consistent with the transcripttomic data, which showed a specific enrichment of pyroptosis-related pathways in HK-2 cells co-treated with LPS and lactate, compared to LPS treatment alone (Figure 1D). Consistently, lactate upregulated cleaved caspase-1 and GSDMD-N expression, enhanced IL-18 and IL-1β secretion, and increased LDH release, which were significantly reversed by 2-DG and OXA (Figure 1E-G). In contrast, inhibitors of ferroptosis (ferrostatin-1, Fer-1), autophagy (3-methyladenine, 3-MA), and apoptosis (Z-VAD-FMK) failed to mitigate lactate-mediated damage exacerbation in LPS-treated HK-2 (HK-2^LPS^) cells (Figure S1G-I). Consistently, *in vivo* experiments revealed that supplementation with lactate remarkably promoted, while 2-DG and OXA obviously inhibited, the pathogenesis of pyroptosis and SA-AKI. This was evidenced by increased cleaved Caspase-1 and GSDMD-N expression, increased IL-18 and IL-1β level (Figure 1H-I), increased serum creatinine (Scr) and blood urea nitrogen (BUN) levels, pronounced tubular epithelial swelling and luminal dilation, elevated KIM-1 expression, upregulated inflammatory cytokines, and reduced survival in CLP-induced SA-AKI mice, which were dramatically rescued by supplementation with 2-DG and OXA (Figure S1J-N).

Notably, the pyroptosis inhibitor Ac-YVAD-cmk significantly ameliorated lactate-aggravated pyroptosis in HK-2^LPS^ cells, restoring cell viability and reducing LDH release and KIM-1 expression (Figure S2A-C). Correspondingly, Ac-YVAD-cmk inhibited Scr, BUN, and KIM-1 expression in CLP mice (Figure S2D-F). Consistent with prior findings, clinical data from 25 SA-AKI patients showed positive correlations between serum lactate concentration and Scr/BUN levels (Figure S2G-H). Analysis of the MIMIC-IV database confirmed that higher lactate levels predicted worsened AKI stages and increased mortality in SA-AKI patients (Figure S2I-J). These findings collectively demonstrated that lactate accelerated pyroptosis of RTEC to promote SA-AKI pathogenesis.

### SPHK1 drives lactate-enhanced pyroptosis of RTEC in SA-AKI via NLRP3 inflammasome activation

**Figure 2:**
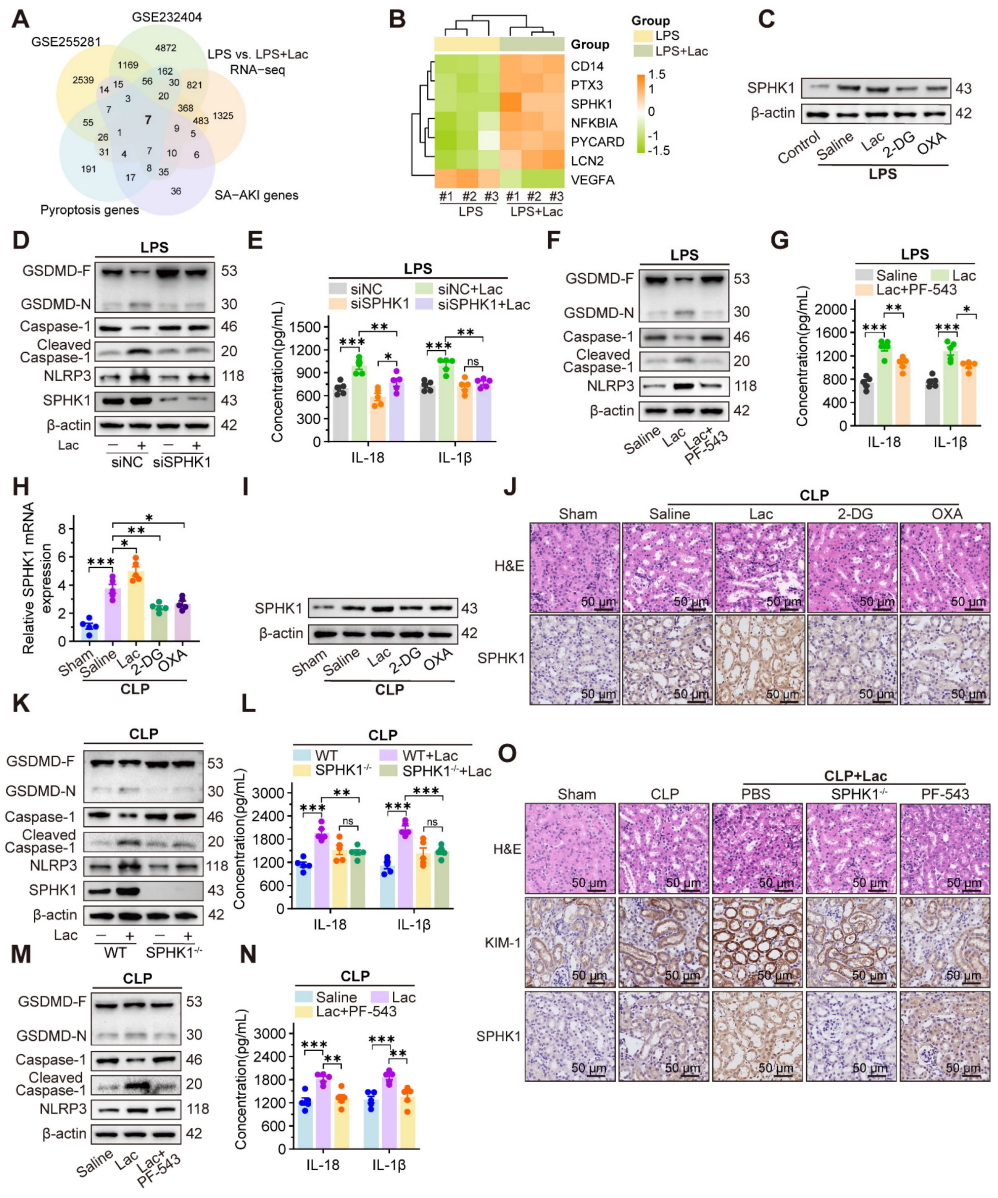
** SPHK1 drives lactate-enhanced pyroptosis of RTEC in SA-AKI via activating NLRP3 inflammasome. (**A) Venn diagram of the intersection of five gene sets identified seven candidate genes linking lactate, pyroptosis, and SA-AKI. (B) Heatmap of candidate genes expression across datasets. (C) Protein levels of SPHK1 in HK-2LPS cells treated with lactate/2-DG/oxamate (n = 5). (D) Protein levels of GSDMD, Caspase-1, Cleaved Caspase-1, NLRP3, and SPHK1 and (E) ELISA analysis of IL-18 and IL-1β levels in HK-2LPS cells transfected with siNC or siSPHK1 under lactate challenge or not (n = 5). (F) Protein levels of GSDMD, Caspase-1, Cleaved Caspase-1, and NLRP3 and (G) ELISA analysis of IL-18 and IL-1β levels in HK-2LPS cells treated with lactate or lactate + PF-543 (n = 5). (H and I) mRNA and protein levels of SPHK1, and (J) representative H&E staining and IHC images of SPHK1 (scale bar = 50 μm) in kidney tissue of CLP mice treated with lactate/2-DG/oxamate (n = 5). (K) Protein levels of GSDMD, Caspase-1, Cleaved Caspase-1, NLRP3, and SPHK1 and (L) ELISA analysis of IL-18 and IL-1β levels in kidney tissue from WT or SPHK1-/- CLP mice under lactate challenge or not (n = 5). (M) Protein levels of GSDMD, Caspase-1, Cleaved Caspase-1, and NLRP3 and (N) ELISA analysis of IL-18 and IL-1β levels in CLP mice treated with lactate or lactate + PF-543 (n = 5). (O) Representative H&E staining and IHC images of KIM-1 and SPHK1 (scale bar = 50 μm) in kidney tissue of CLP mice. Data are mean ± SEM. *p < 0.05, **p < 0.01, and ***p < 0.001; ns, not significant.

To identify potential hub genes mediating lactate-aggravated kidney injury in SA-AKI, we conducted an integrative analysis of five gene sets, including differentially expressed genes (DEGs) from SA-AKI mouse kidney (GSE255281, log_2_FC > 0.585, p < 0.05) and patient blood samples (GSE232404, log_2_FC > 0.585, p < 0.05), SA-AKI associated genes and pyroptosis related genes from the GeneCards database, and our RNA-sequencing in HK-2 cells upon treatment of LPS with or without lactate (log_2_FC > 0.585, p < 0.05). The intersection analysis identified seven candidate genes (Figure 2A), including six upregulated and one downregulated (Figure 2B). Among these genes, SPHK1 was the most elevated gene in HK-2 cells treated with LPS and lactate, which was significantly inhibited by 2-DG and OXA (Figure 2C, Figure S3A).

Given the established role of SPHK1 in inflammation 22-24, we further evaluated SPHK1's involvement in lactate-triggered RTEC pyroptosis. After knocking down SPHK1 with siRNA (Figure S3B-C), the lactate-aggravated pyroptosis in HK-2^LPS^ cells was extensively attenuated, as demonstrated by reduced Caspase-1 cleavage and GSDMD-N formation, and decreased IL-18 and IL-1β secretion (Figure 2D-E). Meanwhile, we identified NLRP3 as the only lactate-upregulated inflammasome component, and its upregulation was abolished by SPHK1 knockdown (Figure 2D, Figure S3D-E). Pharmacological inhibition of SPHK1 using PF-543 similarly restored cell activity by suppressing NLRP3 inflammasome activation and downstream pyroptosis in HK-2^LPS^ cells (Figure 2F-G, Figure S3F). Rescue experiments confirmed that the NLRP3 inducer nigericin (Nig) abolished pyroptosis suppression in SPHK1-silenced HK-2 cells treated with both LPS and lactate (Figure S3G-H).

Furthermore, *in vivo* experiments showed that SPHK1 expression in CLP mice was further upregulated by lactate supplementation but decreased by 2-DG and OXA treatment (Figure 2H-J). Meanwhile, NLRP3-mediated pyroptosis and kidney injury were remarkably alleviated in SPHK1-knockout (SPHK1^-/-^) mice or wild-type (WT) mice treated with PF-543 (Figure 2K-O). This protective effect was abolished by Nig administration (Figure S3I-J). Therefore, these results demonstrated that SPHK1-mediated NLRP3 inflammasome activation was essential for lactate-exacerbated pyroptosis in SA-AKI.

### SPHK1 drives NLRP3-dependent pyroptosis by promoting mitochondrial DNA leakage

**Figure 3:**
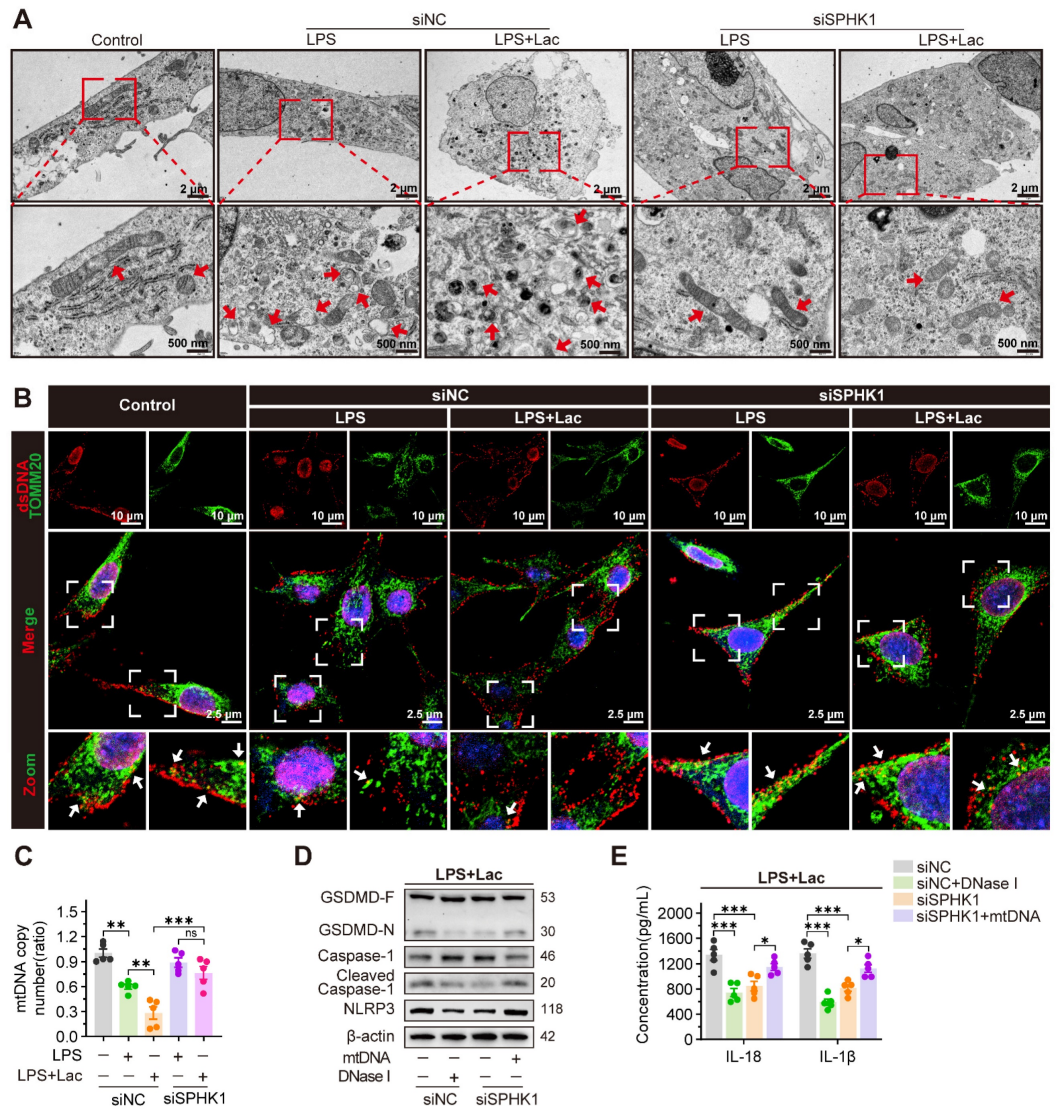
** SPHK1 drives NLRP3-dependent pyroptosis by promoting mitochondrial DNA leakage.** (A) Representative transmission electron microscopy (TEM) images in HK-2LPS cells transfected with siNC or siSPHK1, with or without lactate treatment. Ultrastructural features (mitochondrial integrity) are highlighted (Scale bars = 2 μm, up; 500 nm, down). (B) Representative immunofluorescence images of mtDNA leakage in in HK-2LPS cells transfected with siNC or siSPHK1, with or without lactate treatment (Scale bars =10 μm, up; 20 μm, down). (C) qRT-PCR ananlysis of mtDNA copy number in in HK-2LPS cells transfected with siNC or siSPHK1, with or without lactate treatment (n = 5). (D) Protein levels of GSDMD, Caspase-1, Cleaved Caspase-1, and NLRP3, and (E) ELISA analysis of IL-18 and IL-1β levels in lactate-treated HK-2LPS cells transfected with siNC or siSPHK1, followed by DNase I or mtNDA supplementation (n = 5). Data are mean ± SEM. *p < 0.05, **p < 0.01, and ***p < 0.001; ns, not significant.

Existing evidence demonstrates that mtDNA, as a damage-associated molecular pattern (DAMP), is released into the cytosol under cellular stress to bind and activate NLRP3 25-27. We therefore investigated whether lactate enhanced pyroptosis in RTEC by mediating mtDNA leakage. TEM revealed that LPS induced fragmented mitochondrial cristae in HK-2 cells, which was enhanced by lactate and manifested as mitochondrial swelling and vacuolization. Notably, SPHK1 silencing substantially attenuated these ultrastructural abnormalities even under dual challenge with LPS and lactate (Figure 3A). Morphometric quantification demonstrated that co-stimulation with LPS and lactate decreased total mitochondrial counts, increased damaged mitochondria, and reduced mitochondrial length, all of which were rescued by SPHK1 knockdown (Figure S4A-C).

Using MitoSOX^TM^ (a fluorescent superoxide indicator), we found that lactate further elevated mitochondrial reactive oxygen species (mtROS) levels in HK-2^LPS^ cells, which was effectively attenuated by SPHK1 silencing (Figure S4D). JC-1 staining confirmed that lactate aggravated mitochondrial membrane potential (ΔΨm) collapse in HK-2 cells during LPS exposure, while SPHK1 knockdown stabilized ΔΨm (Figure S4E). Analysis of mitochondrial regulatory networks revealed that lactate downregulated biogenesis/fusion genes (NRF1, TFAM, COX4, SOD2, MFN2) but upregulated the fission marker DRP1 in HK-2^LPS^ cells, which were obviously reversed by SPHK1 knockdown (Figure S4F). These findings collectively indicated that lactate disrupted mitochondrial homeostasis to augment RTEC damage in an SPHK1-dependent manner.

Given the established role of mtDNA in NLRP3 activation, we further examined whether SPHK1 mediated pathological mtDNA release during lactate-induced mitochondrial destabilization. Immunofluorescence showed that co-localization of mtDNA with the mitochondrial marker translocase of outer mitochondrial membrane 20 (TOMM20) was diminished under co-stimulation with LPS and lactate, indicating cytosolic translocation of mtDNA. SPHK1 silencing significantly reduced this redistribution (Figure 3B, Figure S4G). Correspondingly, using a mitochondrial DNA (mtDNA) Monitoring Primer Set in qRT-PCR to measure mtDNA copy number in HK-2 cells, we confirmed progressive mtDNA depletion induced by LPS. This depletion was exacerbated by lactate but rescued by SPHK1 knockdown (Figure 3C).

To establish functional causality between mtDNA leakage and inflammasome activation, we employed DNase I to degrade cytosolic mtDNA. DNase I suppressed NLRP3 activation in HK-2 cells induced by LPS and lactate, as evidenced by diminished Caspase-1 cleavage and GSDMD-N formation and reduced IL-18 and IL-1β secretion (Figure 3D-E). Conversely, SPHK1-silenced cells exhibited attenuated pyroptosis signaling under dual challenge with LPS and lactate, which was reversed by exogenous mtDNA supplementation (Figure 3D-E).

### SPHK1 disrupts mitochondrial homeostasis by inducing ubiquitin-dependent SIRT1 degradation via phosphorylation at Ser47

**Figure 4:**
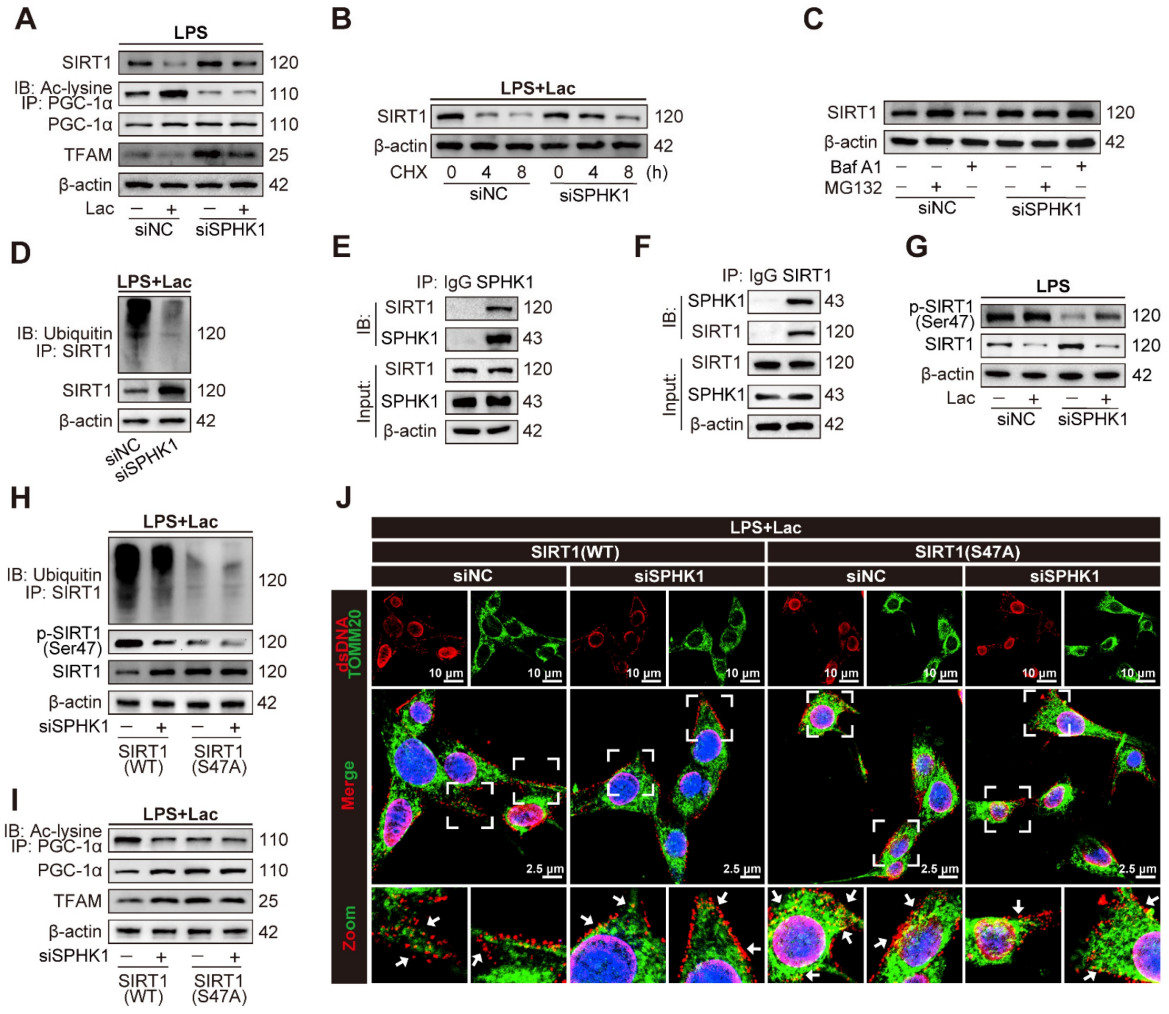
** SPHK1 disrupts mitochondrial homeostasis by inducing ubiquitin-dependent SIRT1 degradation via phosphorylation at Ser47.** (A) Protein levels of SIRT1, PGC-1α acetylation, PGC-1α and TFAM in HK-2LPS cells transfected with siNC or siSPHK1, with or without lactate treatment (n = 5). (B) CHX chase assay demonstrating SIRT1 protein degradation kinetics in lactated-treated HK-2LPS cells transfected with siNC or siSPHK1. (C) SPHK1-induced SIRT1 degradation depends on the ubiquitin-proteasome pathway in lactated-treated HK-2LPS cells. (D) Ubiquitination of SIRT1 in lactated-treated HK-2LPS cells. (E and F) Co-IP detection showed SPHK1-SIRT1 interaction in lactated-treated HK-2LPS cells. (G) Protein levels of SIRT1 phosphorylated at Ser47 and SIRT1 in HK-2LPS cells transfected with siNC or siSPHK1, with or without lactate treatment (n = 5). (H and I) Protein levels of SIRT1 ubiquitination, SIRT1 phosphorylated at Ser47, SIRT1, PGC-1α acetylation, PGC-1α and TFAM and (J) representative immunofluorescence images of mtDNA leakage (Scale bars = 10 μm, up; 2.5 μm, down) in lactated-treated HK-2LPS cells co-transfected with siNC or siSPHK1 and wild-type SIRT1 (SIRT1-WT) or mutant SIRT1 (SIRT1-S47A) (n = 5).

SIRT1 primarily affects mitochondrial function by activating PGC-1α through deacetylation 28-31. Activated PGC-1α acts as a coactivator for mitochondrial transcription factor A (TFAM), which is essential for the maintenance of mtDNA 31. We thus investigated whether SPHK1 regulated mitochondrial homeostasis via the SIRT1/PGC-1α complex in HK-2 cells under co-stimulation of LPS and lactate. qRT-PCR analysis showed that neither lactate nor downregulation of SPHK1 altered mRNA levels of SIRT1 or PGC-1α in HK-2^LPS^ cells (Figure S5A-B). However, co-stimulation with LPS and lactate significantly decreased the mRNA level of TFAM, which was rescued by SPHK1 knockdown (Figure S5C). Western blot analysis showed that lactate significantly decreased SIRT1 and TFAM protein levels in HK-2^LPS^ cells, which was distinctly reversed by SPHK1 knockdown (Figure 4A). Notably, lactate markedly increased acetylation of PGC-1α without altering PGC-1α expression levels in HK-2^LPS^ cells (Figure 4A). These results suggested that lactate might regulate deacetylation-dependent activation of PGC-1α by modulating SIRT1 expression. Further cycloheximide (CHX) chase assays demonstrated that SPHK1 knockdown suppressed SIRT1 degradation in lactated-treated HK-2^LPS^ cells (Figure 4B). We next determined that SIRT1 degradation under LPS/lactate co-treatment occurred primarily via the proteasome pathway, as it was blocked by the proteasome inhibitor MG132 but not autophagy-lysosome inhibitor Bafilomycin A1 (Baf A1) (Figure 4C). Notably, MG132-mediated stabilization of SIRT1 coincided with SPHK1 knockdown, indicating that SPHK1 reduced SIRT1 protein stability by facilitating its degradation via the ubiquitin-proteasome system (Figure 4C). Furthermore, reducing SPHK1 expression decreased ubiquitination of SIRT1 in HK-2 cells exposed to both LPS and lactate (Figure 4D). Consistently, lactate reduced SIRT1 deacetylase activity and PGC-1α nuclear localization in HK-2^LPS^ cells, which was reversed by SPHK1 silencing (Figure S5D-E).

Previous studies have shown an interaction between SPHK1 and SIRT1 32. To examine whether a potential SPHK1-SIRT1 complex functioned in our system, we performed co-immunoprecipitation (co-IP) assays. The results verified a physical interaction between SIRT1 and SPHK1 (Figure 4E-F). Consistently, immunofluorescence co-localization in HK-2^LPS^ cells confirmed significant cytoplasmic interaction between SPHK1 and SIRT1 (Figure S5F). Mechanistically, lactate treatment enhanced this co-localization, which was concomitant with overexpression of SPHK1 and degradation of SIRT1. Based on previous studies indicating that phosphorylation of SIRT1 at Ser47 mediates its inactivation or ubiquitin-dependent degradation 33,34, we hypothesized that SPHK1, as a kinase, might regulate phosphorylation of SIRT1. Consistently, stimulation with lactate remarkably increased Ser47 phosphorylation of SIRT1 in HK-2^LPS^ cells, which was eliminated by SPHK1 knockdown (Figure 4G). Next, we constructed lentiviral plasmids expressing wild-type SIRT1 (SIRT1-WT) and a SIRT1 phosphorylation-defective mutant harboring a serine-to-alanine substitution at Ser47 (SIRT1-S47A). Compared to SIRT1-WT-expressing HK-2^LPS^ cells, ubiquitin-dependent degradation of SIRT1 in SIRT1-S47A-expressing HK-2^LPS^ cells was reduced and could not be further enhanced by SPHK1 silencing (Figure 4H). Reconstitution experiments showed that in HK-2^LPS^ cells, SIRT1-S47A promoted deacetylation of PGC-1α, thereby transcriptionally activating downstream TFAM expression to reduce mtDNA leakage (Figure 4I-J, Figure S5G), which correlated with increased nuclear localization of PGC-1α (Figure S5H). These results demonstrated that the SPHK1-mediated SIRT1/PGC-1α/TFAM axis was critical for lactate-exaggerated mitochondrial collapse in LPS-treated RTEC.

### Lactate increases SPHK1 expression through P300-mediated histone lactylation

**Figure 5:**
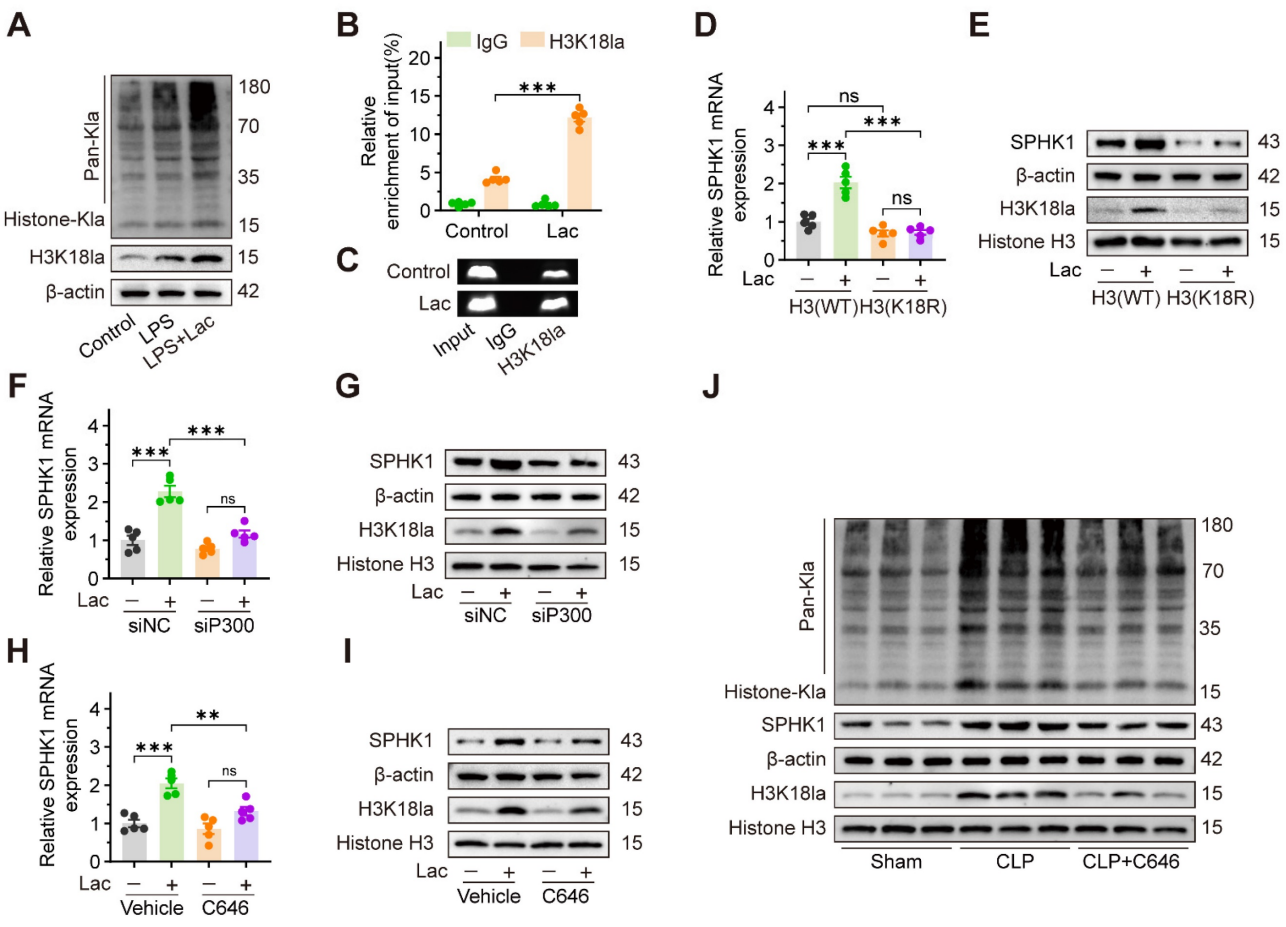
** Lactate increases SPHK1 expression via inducing P300-mediated histone lactylation.** (A) Protein levels of pan-Kla and H3K18la in HK-2 cells treated with LPS and lactate or not (n = 5). (B and C) ChIP-PCR validated the enrichment of H3K18la at the promoter region of SPHK1 in HK-2 cells after lactate stimulation. (D) mRNA levels of SPHK1 and (E) protein levels of SPHK1 and H3K18la in lactate-induced HK-2 cells transfected with wild-type H3 (H3-WT) or mutant H3 (H3-K18R) (n = 5). (F) mRNA levels of SPHK1 and (G) protein levels of SPHK1 and H3K18la in lactate-induced HK-2 cells transfected with siNC or siP300 (n = 5). (H) mRNA levels of SPHK1 and (I) protein levels of SPHK1 and H3K18la in lactate-induced HK-2 cells pretreated with C646 or not (n = 5). (J) Protein levels of SPHK1 and H3K18la in CLP mice pretreated with C646 or not (n = 5). Data are mean ± SEM. *p < 0.05, **p < 0.01, and ***p < 0.001; ns, not significant.

Given accumulating evidence that lactate-induced lactylation modifies protein expression and contributes to pathological conditions, we hypothesized that lactate facilitated SA-AKI progression by epigenetically regulating SPHK1 expression. To investigate the role of lactate-induced lactylation in SA-AKI, we first analyzed global lactylation patterns using pan-Kla antibodies. Western blot analysis revealed a marked accumulation of lactylated proteins in HK-2 cells treated with LPS, which was further enhanced by lactate supplementation. Significantly, a pronounced increase in protein lactylation was observed in the 15-20 kDa range, consistent with the molecular weight of histones (Figure 5A). Cross-species bioinformatics analysis of the Compendium of Protein Lysine Modifications (CPLM) database further identified conserved lactylation sites on histones in both humans and mice (Figure S6A-B). Given that histone H3 lysine 18 lactylation (H3K18la) is a known transcriptional regulator 13,35, we focused our subsequent investigation on this specific modification. Analysis of Gene Expression Omnibus (GEO) datasets (GSE242018 and GSE229154) revealed significant H3K18la enrichment at the SPHK1 promoter (Figure S6C-D). Consistent with these findings, lactate distinctly increased H3K18la levels (Figure 5A). Meanwhile, chromatin-immunoprecipitation (ChIP) assays further confirmed that H3K18la was enriched at the SPHK1 promoter (Figure 5B-C).

To further validate whether lactate-induced H3K18la regulated SPHK1 transcription, HK-2 cells were transfected with lentiviral constructs expressing either wild-type histone H3 (H3-WT) or a mutant form in which lysine 18 was replaced with arginine (H3-K18R, a mutation that prevents lactylation at this site). As expected, lactate increased both mRNA and protein expression levels of SPHK1 in HK-2 cells transfected with H3-WT, but not in HK-2 cells transfected with H3-K18R (Figure 5D, E). Since the lysine acetyltransferase P300 catalyzes lactyl group transfer from lactyl-CoA to histones, we investigated its role in lactate-induced H3K18la in HK-2 cells. After knocking down P300 with siRNA (Figure S6E-F), lactate failed to enhance SPHK1 expression in HK-2 cells (Figure 5F-G). Moreover, C646, a pharmacological P300 inhibitor, also impeded lactate-induced SPHK1 overexpression in HK-2 cells (Figure 5H-I). Consistently, C646 significantly attenuated H3K18la levels and SPHK1 expression in kidney tissue of CLP mice (Figure 5J). Together, these results demonstrated that lactate epigenetically regulated SPHK1 transcription through P300-mediated H3K18la, thereby promoting SA-AKI pathogenesis.

### NAD^+^ emerges as a therapeutic candidate targeting SPHK1-driven kidney injury by activating SIRT1-mediated delactylation

**Figure 6:**
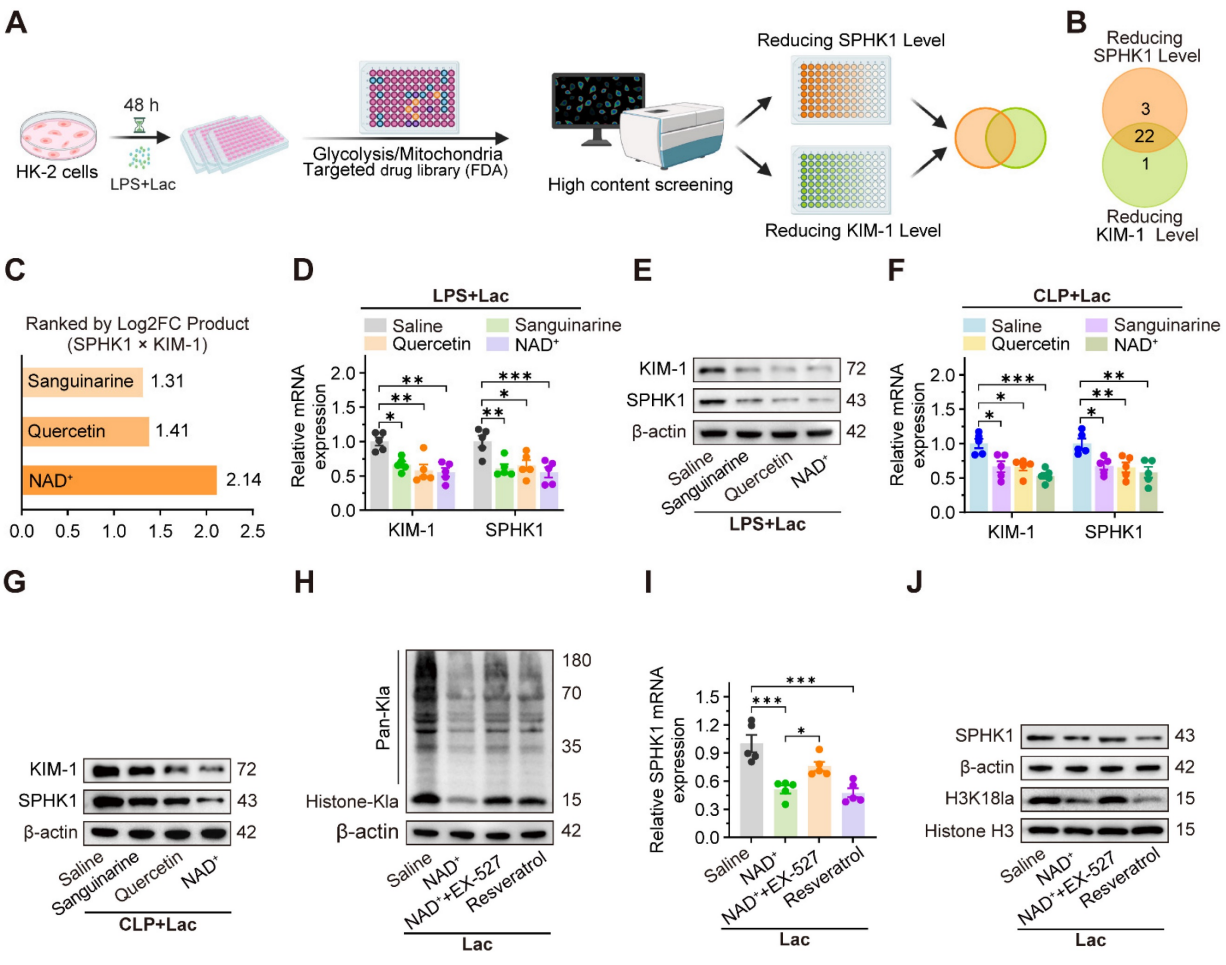
** NAD+ emerges as a therapeutic candidate targeting SPHK1-driven kidney injury via activating SIRT1-mediated delactylation.** (A) Schematic of high-throughput drug screening workflow in lactate-treated HK-2LPS cells. (B) Venn diagram identified 22 compounds that coordinately downregulate SPHK1 and KIM-1. (C) Top 3 candidates (NAD+, quercetin, and sanguinarine) ranked by efficacy. (D and E) mRNA and protein levels of KIM-1 and SPHK1 in lactate-treated HK-2LPS cells pretreated with sanguinarine/quercetin/NAD+ (n = 5). (F and G) mRNA and protein levels of KIM-1 and SPHK1 in kidney tissue of CLP mice subjected to lactate pretreated with sanguinarine/quercetin/NAD+ (n = 5). (H) Protein levels of pan-Kla in lactate-treated HK-2 cells pretreated with NAD+, NAD+ + EX-527, or resveratrol (n = 5). (I) mRNA levels of SPHK1 and (J) protein levels of SPHK1 and H3K18la in lactate-treated HK-2 cells pretreated with NAD+, NAD+ + EX-527, or resveratrol (n = 5). Data are mean ± SEM. *p < 0.05, **p < 0.01, and ***p < 0.001; ns, not significant.

Given the central role of SPHK1 in lactate-induced pyroptosis of RTEC, we conducted a high-throughput drug screen to identify compounds that simultaneously suppressed the expression of both SPHK1 and the kidney injury biomarker KIM-1. Using a library of 400 Food and Drug Administration (FDA)-approved drugs targeting glycolytic or mitochondrial pathways, we quantified SPHK1 and KIM-1 expression by high-content imaging in HK-2 cells exposed to LPS and lactate (Figure 6A). Initial screening (Figure S7A-D) identified 22 compounds that coordinately downregulated both SPHK1 and KIM-1 expression (Figure 6B). Among these, NAD⁺, quercetin, and sanguinarine emerged as the top three candidates (Figure 6C), which was further confirmed by qRT-PCR and western blot assays in lactate-treated HK-2^LPS^ cells. Among the three agents, NAD⁺ demonstrated the highest inhibitory efficacy (Figure 6D-E, Figure S7F), as confirmed in the CLP model (Figure 6F-G).

Since SIRT1, an NAD⁺-dependent deacetylase, has been identified as a potential “eraser” of histone lactylation 36, we investigated whether NAD⁺ mediated SPHK1 reduction through SIRT1-dependent delactylation. As expected, lactate-induced global histone lactylation in HK-2 cells was significantly reversed by NAD⁺ and the SIRT1 activator resveratrol, which was abolished by the SIRT1 inhibitor EX-527 (Figure 6H). Consistently, lactate-induced upregulation of SPHK1 and H3K18la levels in HK-2 cells was also markedly reduced by NAD⁺ and resveratrol, which was abolished by EX-527 (Figure 6I-J). These findings revealed a negative feedback loop between SPHK1 and SIRT1, positioning NAD⁺ as a potential therapeutic agent for SA-AKI by suppressing lactate-mediated SPHK1 expression.

### PF-543 and NAD^+^ synergistically alleviate the progression of SA-AKI

**Figure 7:**
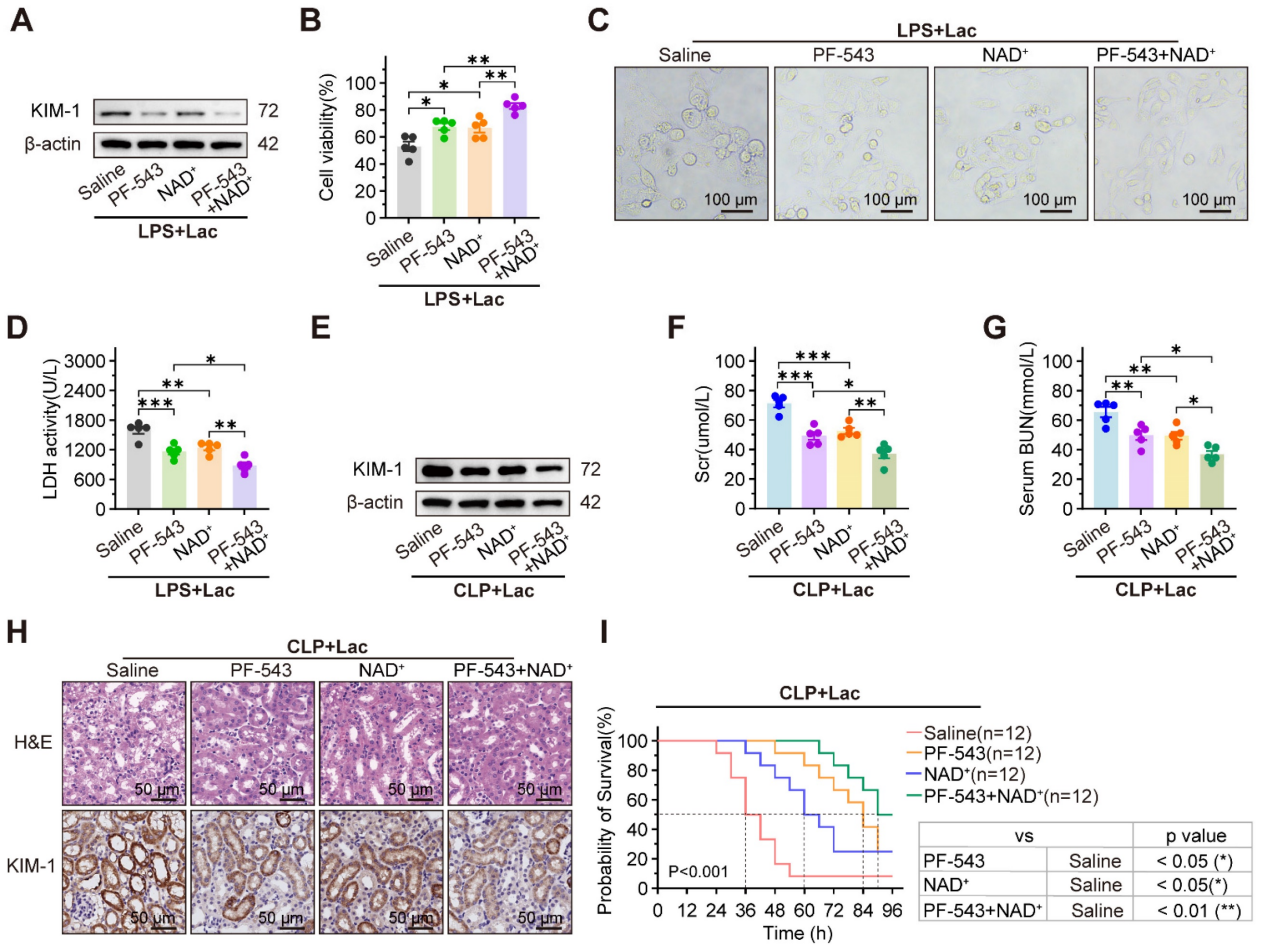
** PF-543 and NAD+ synergistically alleviate progression of SA-AKI.** (A) Protein levels of KIM-1, (B) cell viability, (C) representative morphological features of pyroptosis (bright-field, scale bar = 100 μm) and (D) LDH release in lactated-treated HK-2LPS cells pretreated with PF-543, NAD+, or PF-543 + NAD+ (n = 5). (E) Protein levels of KIM-1, (F) Scr, (G) BUN, (H) representative H&E staining and IHC images of KIM-1 in kidney tissue of CLP mice subjected to lactate pretreated with PF-543, NAD+, or PF-543 + NAD+ (n = 5). (E) Survival curves of CLP mice subjected to lactate pretreated with PF-543, NAD+, or PF-543 + NAD+ (n = 12). Data are mean ± SEM. *p < 0.05, **p < 0.01, and ***p < 0.001; ns, not significant.

To assess the synergistic effects of the SPHK1 inhibitor PF-543 and the SIRT1 activator NAD⁺, both cells and mice sepsis models were treated with PF-543 and/or NAD⁺. In lactate-treated HK-2^LPS^ cells, elevated KIM-1 expression, reduced cell viability, pyroptosis-associated morphological damage, and increased LDH release were significantly reversed by PF-543 or NAD⁺ monotherapy, with further suppression observed with combined treatment (Figure 7A-D). Consistent with cellular findings, combined administration of the SPHK1 inhibitor PF-543 and NAD⁺ in CLP mice produced synergistic benefits, more effectively improving renal function, reducing injury markers and histopathological damage, and prolonging survival than either agent alone (Figure 7E-I). These results demonstrated that the combination of SPHK1 inhibition and SIRT1 activation is a potent therapeutic strategy for SA-AKI.

## Discussion

While pyroptosis is confirmed as a vital mechanism leading to tubular injury in SA-AKI pathogenesis, and is known to accelerate disease progression and correlate with poor outcomes, it remained unclear whether lactate directly affected pyroptosis. This study provided the first evidence that lactate exacerbated SA-AKI by promoting RTEC pyroptosis via an epigenetic-metabolic mechanism. Mechanistically, lactate-derived H3K18la enrichment at the SPHK1 promoter, mediated by P300, activated SPHK1 transcription. SPHK1 subsequently phosphorylated SIRT1 and mediated its degradation via a ubiquitin-dependent mechanism. The loss of SIRT1 deacetylase activity suppressed PGC-1α transcripttional activity, impairing mitochondrial biogenesis and antioxidant responses. This mitochondrial dysfunction triggered mtDNA leakage and NLRP3 inflammasome activation, resulting in pyroptosis. Interestingly, we found that SIRT1 functioned as a delactylase, removing H3K18la to suppress SPHK1 expression, thereby forming a negative feedback loop. Interestingly, enhancing this loop via NAD^+^, particularly in combination with the SPHK1 inhibitor PF-543, synergistically attenuated SA-AKI by simultaneously targeting histone lactylation and SPHK1 activity. This dual intervention provided a promising approach targeting the interaction between metabolism, epigenetics, and inflammation in SA-AKI.

Pyroptosis, mediated by NLRP3 inflammasome activation and gasdermin-dependent lysis, is a key driver of tubular damage in SA-AKI. Previous studies have shown that caspase-11/GSDMD-dependent pyroptosis triggers tubular injury and increased urinary IL-18 levels in AKI 19. Sun et al. reported that USF2 worsened SA-AKI by this process through the TGF-β/Smad3/NLRP3/Caspase-1 axis 8. However, the influence of metabolic reprogramming on pyroptosis remained unexplored. Our findings revealed that lactate directly aggravated LPS-induced pyroptosis in RTEC, thereby accelerating SA-AKI progression. Consistent with previous research associating lactate with sepsis-induced multi-organ dysfunction 11,12, clinical cohort data and MIMIC-IV analysis confirmed that hyperlactatemia correlated with SA-AKI severity and mortality. These findings broadened our comprehension of lactate's role in pyroptosis during SA-AKI and highlighted the influence of the metabolic microenvironment on inflammatory progression. Notably, lactate exacerbated SA-AKI only in the cases of pre-existing injury, as evidenced by minimal RTEC damage when exposed to high-concentration lactate alone (20 mM *in vivo*, 10 mM *in vitro*). This suggests that intact metabolic enzyme activity can effectively clear lactate under physiological conditions, which is consistent with findings reported by An et al 11. In addition to its metabolic role, lactate-mediated histone lactylation is increasingly recognized for its involvement in organ injury during sepsis. We identified a mechanistic link between lactate and pyroptosis through the epigenetic regulation of SPHK1. Lactate promoted P300-mediated H3K18la enrichment at the SPHK1 promoter, leading to increased transcription, which was confirmed by H3-K18R mutation and P300 inhibition. SPHK1 then initiated a cascade of post-translational modifications (PTMs). It phosphorylated SIRT1 at Ser47, causing its degradation and resulting in PGC-1α hyperacetylation and inactivation. This caused mtDNA leakage and NLRP3 inflammasome activation. Our work thus identified a three-part PTM axis (lactylation-phosphorylation-acetylation) that collectively regulated lactate-induced pyroptosis in SA-AKI.

Interestingly, high-throughput screening targeting SPHK1 and KIM-1 identified NAD⁺ as a therapeutic candidate. We defined a self-enhancing SPHK1-SIRT1 loop, in which SIRT1 combined metabolic stress and mitochondrial dysfunction, operating as both a deacetylase and lactylation "eraser" 36. NAD⁺ suppressed SPHK1 expression by activating SIRT1-mediated delactylation, thereby forming a negative feedback loop between SPHK1 and SIRT1. Lactate disrupted this protective loop to promote pyroptosis, and NAD⁺ restored it. Building on this mechanistic insight, we pursued a dual-targeting strategy aimed at comprehensively breaking the lactate-driven vicious cycle. Notably, NAD⁺ activated SIRT1 to reduce H3K18la and suppress SPHK1 expression, and PF-543 inhibited SPHK1 kinase activity to rescue SIRT1/PGC-1α function. This combined strategy simultaneously addressed epigenetic dysregulation and signaling hyperactivation.

Although this work has improved the comprehension of metabolic microenvironment regulation of pyroptosis in SA-AKI, there are several limitations that need to be considered. First, our focus on histone lactylation prompts the question of whether lactate influences pyroptosis through lactylation of other key non-histone proteins. Second, even though NAD⁺ and PF-543 showed effectiveness in preclinical studies 37, clinical translation requires overcoming pharmacokinetic challenges, particularly rapid NAD⁺ degradation. Emerging solutions, such as nanoparticle-based NAD⁺ delivery or stabilized precursors, could overcome this barrier and form the basis of next-generation combination nanotherapies 38. A critical caveat is the paradoxical effect we observed with the NAD⁺ precursor nicotinamide (NAM), which unexpectedly upregulated SPHK1 and KIM-1, likely due to dose-dependent inhibition of nicotinamide phosphoribosyltransferase (NAMPT). This highlights the importance of optimized NAD⁺ delivery methods to prevent counterproductive effects.

In summary, this study revealed that lactate exacerbates SA-AKI by promoting RTEC pyroptosis via H3K18la-mediated SPHK1 upregulation, which disrupted mitochondrial homeostasis through SIRT1/PGC-1α inhibition, leading to NLRP3 inflammasome activation. The combination of NAD^+^ and SPHK1 inhibitors effectively reduced SA-AKI by restoring SIRT1-mediated delactylation and mitochondrial function. This dual-targeting strategy presents a promising therapy for SA-AKI.

## Conclusion

In summary, this study reveals that lactate exacerbates SA-AKI by promoting RTEC pyroptosis through an H3K18la-mediated SPHK1-SIRT1 feedback loop. Lactate-driven H3K18la transcriptionally upregulates SPHK1, which triggers SIRT1 phosphorylation and degradation. This collapse of SIRT1 function disrupts mitochondrial integrity via PGC-1α hyperacetylation and mtDNA leakage, ultimately activating NLRP3 inflammasome-driven pyroptosis. Conversely, SIRT1 acts as a delactylase, forming a negative feedback loop to suppress SPHK1 expression. Drug screening identified NAD^+^ as as a potent inhibitor of SPHK1 and KIM-1 by restoring SIRT1 activity, while the combination of NAD^+^ and PF-543 synergistically attenuates SA-AKI progression. These findings highlight lactate-induced epigenetic-metabolic crosstalk as a key driver of pyroptosis in SA-AKI and propose that the combination of NAD^+^ supplementation and SPHK1 inhibition represents a promising method to mitigate SA-AKI.

## Methods

### Human blood samples

Human blood sample collection was conducted under a protocol approved by the Ethics Committee of Union Hospital, Tongji Medical College, Huazhong University of Science and Technology (UHCT-IEC-SOP-016-03-01) with patient informed consent. From each sepsis patient, 10 mL of peripheral venous blood was drawn within 1 h of ICU admission. Patient demographics and clinical data are provided in Table S1 (Supporting Information).

### Mice

C57BL/6 mice (Vital River Laboratory) and SPHK1-/- mice (gift from Prof. Zhou Hong, Anhui Medical University) were housed under standard conditions. The SA-AKI model was established via cecal ligation and puncture (CLP). Under pentobarbital anesthesia, a midline laparotomy was performed. The cecum was ligated 15 mm from its apex and then punctured once with a 20-gauge needle to extrude minimal fecal content before being returned to the abdominal cavity. Sham mice underwent laparotomy without CLP. All mice received postoperative saline and supportive care. To evaluate the role of lactate, mice received an intraperitoneal injection of sodium lactate (NaLa, 5 M, 1g/kg) or saline immediately after CLP. In separate experiments, mice were treated with oxamate (MCE, HY-W013032A, 0.5 g/kg), 2-DG (MCE, HY-D13966, 0.5 g/kg), AC-YVAD-CMK (MCE, HY-16990, 10 mg/kg), Nigericin (MCE, HY-127019, 1 mg/kg), PF-543 (MCE, HY-15425, 10 mg/kg), C646 (MCE, HY-13823, 10 mg/kg), Sanguinarine (MCE, HY-N0052, 10 mg/kg), Quercetin (MCE, HY-18085, 50 mg/kg), NAD^+^ (MCE, HY-B0445, 100 mg/kg), EX-527 (Selleck, S1541, 10 mg/kg), and Resveratrol (Selleck, S1396, 50 mg/kg). At indicated times, samples were collected and either fixed in 4% paraformaldehyde or flash-frozen at -80°C. Whole blood was centrifuged (3000 rpm, 15 min) to isolate serum, which was then aliquoted and stored at -80°C.

### Cell culture and treatments

HK-2 cells (CVCL_0302) were purchased from Procell Life Science&Technology Co., Ltd. (CL-0109, Wuhan, China). The HK-2 human renal tubular epithelial cell line, a validated model for SA-AKI studies 11,39, was cultured in DMEM/F-12 medium with 10% FBS under standard conditions (37°C, 5% CO₂). All cell lines were tested and confirmed free of contamination prior to use. HK-2 cells were stimulated with different concentrations of NaLa (MCE, HY-B2227B) for 24 h, and 5 mM was selected as the pre-treatment concentration of combination with LPS (MCE, HY-D1056, 10 μg/mL). In separate experiments, HK-2 cells were treated with 2-DG (MCE, HY-D13966, 5 mM), oxamate (MCE, HY-W013032A, 5 mM), Fer-1 (Selleck, S7243, 10 μM), 3-MA (MCE, HY-19312, 5 mM), Z-VAD-FMK (MCE, HY-16658B, 20 μM), AC-YVAD-CMK (MCE, HY-16990, 40 μM), Nigericin (MCE, HY-127019, 2 μg/mL), PF-543 (MCE, HY-15425, 100 nM), C646 (MCE, HY-13823, 10 μM), Sanguinarine (MCE, HY-N0052, 2 μM), Quercetin (MCE, HY-18085, 4 μM), NAD^+^ (MCE, HY-B0445, 200 μM), EX-527 (Selleck, S1541, 10 μM), and Resveratrol (Selleck, S1396, 10 μM).

### Cell viability assay

Cell viability was determined using the CCK-8 assay (Beyotime). Briefly, we seeded HK-2 cells into 96-well plates (5×10³ cells/well) and serum-starved them overnight. Cells were then treated as indicated. To measure viability, we added 10 μL of CCK-8 reagent per well, incubated the plates at 37°C for 2 h, and quantified the absorbance at 450 nm.

### LDH release assay

LDH release, an indicator of cytotoxicity, was quantified with a dedicated assay kit (Beyotime). After plating and serum starvation (as above), HK-2 cells were treated. The LDH release reagent was then added. Following incubation, supernatants were collected for enzymatic activity measurement per the manufacturer's protocol.

### Measurement of lactate levels

We quantified lactate using a commercial kit (Beyotime). For serum, clotted blood was centrifuged to collect supernatant. Kidney tissue was homogenized and centrifuged to obtain a clear lysate. For HK-2 cells, intracellular lactate was extracted via lysis/centrifugation, while extracellular lactate was measured directly from medium. All prepared supernatants were analyzed immediately per the protocol.

### Biochemical indicator detection protocol

We assessed renal function by measuring Scr and BUN using commercial kits (Beyotime). Scr was quantified by incubating 20 μL serum with Amplex Red reagent at 37 °C for 30 min in the dark, followed by absorbance measurement at 570 nm. BUN levels were determined under identical incubation conditions, using 50 μL serum treated with enzyme solution and working reagent, with absorbance read at 670 nm. All concentrations were calculated from standard curves.

### Transmission electron microscopy (TEM)

Mitochondrial ultrastructure in HK-2 cells was examined using TEM. Cells were processed for TEM by sequential fixation (2.5% glutaraldehyde, then 1% osmium tetroxide), ethanol dehydration, and epoxy resin embedding. Ultrathin sections were stained with uranyl acetate and lead citrate and examined under a Hitachi H7650 microscope. Morphological changes in mitochondria were measured in five random fields per sample. Experiments were confirmed with three independent replicates, and representative images were selected for presentation.

### mtDNA extraction

Mitochondrial DNA (mtDNA) was isolated from HK-2 cells (5×10⁶ cells per sample) using a dedicated kit (Abcam). Cells were collected by centrifugation, washed with ice-cold PBS, and then processed according to the manufacturer's instructions for mitochondrial fractionation and DNA purification. After 10-min incubation on ice, cells were homogenized with 50-100 strokes in a pre-cooled Dounce grinder. The efficiency of homogenization was confirmed microscopically (>80% of nuclei lacking intact plasma membranes). Following homogenization, nuclei and debris were removed by low-speed centrifugation (1,200 × g, 10 min, 4 °C). Mitochondria were subsequently pelleted from the supernatant by centrifugation (10,000 × g, 30 min, 4°C) at 10,000 × g for 30 min at 4°C. Mitochondria were lysed in 30 μL of Lysis Buffer III and digested with 5 μL of Enzyme Mix for 60 min at 50 °C. mtDNA was precipitated with 35 μL 5 M ammonium acetate and 140 μL of ethanol for 30 min at -20 °C, washed twice with 70% ethanol, and resuspended in 20 μL of TE Buffer. DNA concentration was quantified spectrophotometrically (NanoDrop), and integrity was confirmed by 0.8% agarose gel electrophoresis (16-20 kb band).

### Lentivirus infection

Mutant constructs of SIRT1-S47A and H3C1-K18R were generated by PCR-based mutagenesis using specific primers (GeneChem, Shanghai). The full-length sequences of SIRT1-WT (RefSeq NM_012238.5), SIRT1-S47A, H3C1-WT (RefSeq NC_000006.12), and H3C1-K18R were individually cloned into lentiviral vector GV341 (Ubi-MCS-3FLAG-SV40-puromycin). Recombinant LV5 plasmids were transfected into HK-2 cells using Lipofectamine 3000 (Invitrogen), followed by puromycin selection (HY-B1743A, MCE). Functional assays were conducted 48 h after transfection.

### Chromatin immunoprecipitation assay

Chromatin immunoprecipitation (ChIP) was performed using a commercial kit (Beyotime). Briefly, chromatin from cross-linked HK-2 cells was sonicated and immunoprecipitated overnight at 4°C with the indicated antibody (normal IgG served as control). After capturing immune complexes with Protein A/G magnetic beads and washing, bound DNA was eluted. Enrichment at specific promoter regions was quantified by qPCR using primers listed in Table S5 (Supporting Information).

### Compound library screening

To identify modulators of the lactate-SPHK1 axis, we screened a compound library targeting glycolysis and mitochondrial pathways (Selleck). For the screen, HK-2 cells were plated in 96-well plates, serum-starved overnight, and then exposed to individual library compounds or vehicle control. The fluorescence intensity of the two key molecules was monitored by a high-content confocal imaging system in which 15 non-overlapping fields of view per well were captured at 40 × magnification. All compounds were screened at a concentration of 10 nM to assess their effects on the cells, and all wells were stained evaluate the compounds' influence on these specific markers. Three replicate wells were conducted for each compound. Compile a detailed list of the compounds screened, including their concentrations and observed effects, as outlined in Supplemental Table S6. The initial selection and prioritization of candidates were based on their ability to concurrently downregulate the expression of two key markers: the core molecule SPHK1 and the established kidney injury marker KIM-1. Candidates were ranked according to the product of the fold-change decrease in the expression of both SPHK1 and KIM-1, leading to the selection of the top 3 candidates for further functional validation.

### SIRT1 activity measurement

SIRT1 activity in HK-2 cells lysates was quantified using the fluorometric SIRT1 Activity Assay Kit (ab156065, Abcam) per manufacturer's protocol. Cells were lysed in ice-cold lysis buffer (containing 20 mM Tris-HCl pH 7.5, 250 mM NaCl, 1% Triton X-100, 1 mM DTT, 1 mM EDTA/EGTA) without protease inhibitors, followed by centrifugation to collect supernatants. Reactions containing assay buffer, 20 μM fluorogenic substrate, 200 μM NAD^+^, Developer solution, and cell lysate (5-20 μg protein) were incubated at room temperature. Fluorescence intensity (Ex/Em = 350/460 nm) was kinetically recorded every 1-2 min for 30-60 min. Activity was calculated as the slope of fluorescence increase (RFU/min) after subtracting the background rate from "No Enzyme" controls (lysis buffer only). All measurements were performed in duplicate.

### Mitochondrial superoxide detection

We measured mitochondrial superoxide levels by staining HK-2 cells with 500 nM MitoSOX™ Red reagent for 30 min at 37°C (protected from light). Following three washes with warm HBSS, images were captured without delay. Fluorescence was detected at 396/610 nm (excitation/emission) using a fluorescence microscope.

### Mitochondrial membrane potential assay

Mitochondrial membrane potential in HK-2 cells was assessed using the JC-1 Assay Kit (C2003, Beyotime). Cells were incubated with JC-1 staining working solution (prepared by 1:200 dilution of 200× JC-1 in JC-1 staining buffer) for 20 min at 37 °C. After removal of the staining solution, cells were washed twice with JC-1 staining buffer. For fluorescence microscopy, cells were maintained in culture medium and immediately imaged. The ratio of red (aggregates, at 525/590 nm, high potential) to green (monomers, at 490/530 nm, low potential) fluorescence was quantified to reflect changes in membrane potential.

### Subcellular protein fractionation

Cytoplasmic and nuclear proteins were fractionated from HK-2 cells using a commercial extraction kit (Boster). Briefly, cells were lysed in a two-step process to sequentially isolate cytoplasmic and nuclear fractions according to the manufacturer's protocol. All protein fractions were aliquoted and stored at -80°C for subsequent Western blot analysis.

### Data extraction from MIMIC-IV database

The patient cohort was obtained from MIMIC-IV database, which contains comprehensive electronic medical records of more than 50,000 ICU admissions. As the data has been de-identified, this study is exempt from the informed consent requirement, and all authors obtained the necessary data usage certifications. Eligible patients were adults (≥18 years) on their first ICU admission (>24 hours) who met Sepsis-3 criteria and developed AKI (by KDIGO criteria) within seven days. Exclusions comprised pre-existing end-stage renal disease, CKD stage 5, and cases with incomplete records. For included patients, we extracted demographics, clinical parameters, and outcomes from the database. Missing data were imputed using appropriate methods based on the extent of missingness, and outliers were removed if clinically implausible. Feature selection was performed to reduce multicollinearity and retain clinically relevant variables. Approval for the MIMIC-IV database was granted by the Institutional Review Board (IRB) at the Massachusetts Institute of Technology (MIT). Among the authors of this study, Hongda Wang has secured access to the database and taken charge of data extraction.

### Western blot analysis

Proteins were extracted using RIPA buffer with protease inhibitors. Following protein quantification (BCA assay), equal amounts were separated by SDS-PAGE and transferred to PVDF membranes. After blocking the membranes with TBST buffer containing 5% skim milk or 5% BSA, the membranes were incubated at 4 °C overnight with primary antibodies against SPHK1 (BA2865, Boster), KIM-1 (NBP1-76701, Novus), GSDMD (39754, CST), Caspase-1 (83383, CST), H3K18la (PTM-1427RM, PTM Bio), Cleaved Caspase-1 (89332, CST), P300 (ab275378, Abcam), Cleaved Caspase-1 (4199, CST), NLRP3 (P60622R3, Abmart), SIRT1 (13161-1-AP, Proteintech), NLRC4 (PB0658, Boster), PGC-1α (66369-1-Ig, Proteintech), Acetylated-Lysine (9441, CST), p-SIRT1 (SAB4301426, MilliporeSigma), Pan-Kla (PTM-1401RM, PTM Bio), Pan-Ubiquitin (PTM-1124RM, PTM Bio), AIM2 (20590-1-AP, Proteintech), NLRP1 (12256-1-AP, Proteintech), Histone H3 (PTM-1002RM, PTM Bio) and β-actin (AC026, Abclonal). Following secondary antibody incubation, blots were developed with ECL and imaged. Protein band intensities were quantified using ImageJ software for comparative analysis.

### Co-immunoprecipitation

For co-immunoprecipitation, HK-2 cells were lysed in NP-40 buffer supplemented with protease, phosphatase, and deacetylase inhibitors. After centrifugation, cleared lysates were incubated overnight 4 °C with anti-SPHK1 (10670-1-AP, Proteintech), anti-SIRT1 (13161-1-AP, Proteintech), or anti-PGC-1α (66369-1-Ig, Proteintech), with control IgG for background subtraction. Following incubation with antibodies, complexes were captured on Protein A/G beads, washed extensively with lysis buffer, and eluted by heating in SDS-PAGE buffer prior to immunoblot analysis. Finally, Western blot detection was performed using the above-mentioned specified antibodies.

### Measurement of IL-1β and IL-18

IL-18 and IL-1β levels were measured in mouse kidney tissue lysates and HK-2 cell medium using species-specific ELISA kits (Boster). Clear supernatants were obtained from kidney tissues by homogenization followed by centrifugation (12,000 × g, 10 min, 4°C). For cell culture media, a brief centrifugation (1,000 × g, 10 min, 4°C) was performed to remove debris before analysis. Mouse IL-1β (EK0394) and IL-18 (EK0433) kits were used for renal samples; human IL-1β (EK0392) and IL-18 (EK0864) kits for HK-2 cells media. Samples/standards were added to pre-coated plates, incubated with biotinylated antibodies and ABC reagent, followed by TMB substrate. Absorbance was read at 450 nm. Concentrations were calculated from standard curves. Experiments included three independent replicates.

### siRNA transfection

Small interfering RNAs (siRNAs) targeting the SPHK1 gene and P300 gene was designed and synthesized by GenePharma. The above siRNAs were transfected into cells *in vitro* according to the instructions of Lipofectamine 3000 transfection reagent (Invitrogen). After transfection for 48 h, the silencing efficiency of siRNA on target genes was verified by qRT-PCR and Western blot. The sequences of the siRNAs used in this experiment are detailed in Table S3 (Supporting Information).

### RNA isolation and quantitative real-time PCR

Total RNA was extracted from HK-2 cells or kidney tissue using an RNA extraction kit (Takara, Japan). mRNA levels were quantified by real-time PCR using SYBR Green chemistry (Takara).The mRNA expression levels of the target genes were normalized and adjusted using β-actin as the reference gene. Details of the primer sequences used in this experiment are provided in Table S4 (Supporting Information).

### Histology and immunohistochemistry

Paraffin-embedded tissues were sectioned (6 µm) for H&E staining and immunohistochemistry. IHC sections were processed through deparaffinization, rehydration, peroxidase blocking, and antigen retrieval steps. After being blocked with 10% donkey serum, the sections were incubated overnight at 4°C with primary antibody anti-SPHK1 (BA2865, Boster) and anti-KIM-1 (NBP1-76701, Novus). Signal detection was achieved by sequential incubation with biotinylated secondary antibodies and DAB substrate (DAKO kit). Sections were then counterstained with hematoxylin and evaluated by light microscopy (Olympus).

### Immunofluorescence

HK-2 cells plated on confocal dishes were fixed (4% PFA, 15 min) and permeabilized (0.2% Triton X-100, 10 min) as part of the standard immunofluorescence staining protocol prior to antibody labeling. The cells were blocked with 5% normal goat serum and 1% BSA for 1 h, primary antibodies - anti-TOMM20 (ab78547, Abcam) and anti-dsDNA (ab27156, Abcam) - were added, and incubation was carried out overnight at 4°C. Following PBS washes, samples were incubated with fluorescent secondary antibodies (Alexa Fluor 488 or 594) for 2 h at room temperature in the dark. After counterstaining nuclei with DAPI, samples were mounted in a fluorescent anti-quenching medium and visualized with a Nikon confocal laser scanning microscope.

### Identification of DEGs by combining several gene sets

The gene expression datasets (GSE232404 and GSE255281) were retrieved from the NCBI GEO database. Pyroptosis genes and SA-AKI genes were downloaded from the Genecards database. HK-2 cells treated with LPS alone or LPS combined with lactate were subjected to transcriptome sequencing. The R software package was used to analyze the gene set, transform and filter the unqualified data, and correct, standardize and transform the data. Processed expression data were analyzed for differential expression with the R/Bioconductor package Limma. Genes with an FDR-adjusted p-value below 0.05 were considered significantly differentially expressed.

### Statistical analysis

All statistical analyses were performed using GraphPad Prism software (version 9.0). Data are presented as mean ± standard error of the mean (SEM), with the sample size (n) detailed in the figure legends. After verifying normality and equal variance, group differences were analyzed. Student's t tests (two-tailed) were applied to determine the differences between the two groups. The one-way ANOVA test was applied to determine three or more groups, followed by Tukey's post hoc test. Chi-square (x^2^) test was applied to determine correlations between categorical variables, Pearson's correlation analysis was applied to determine correlations between continuous variables. Kaplan-Meier analysis with log-rank tests were applied to compare survival differences. Each data group underwent a variation estimate. *p < 0.05, **p < 0.01, and ***p < 0.001, with ns meaning not significant.

## Supplementary Material

Supplementary figures and tables.

## Figures and Tables

**Figure 8:**
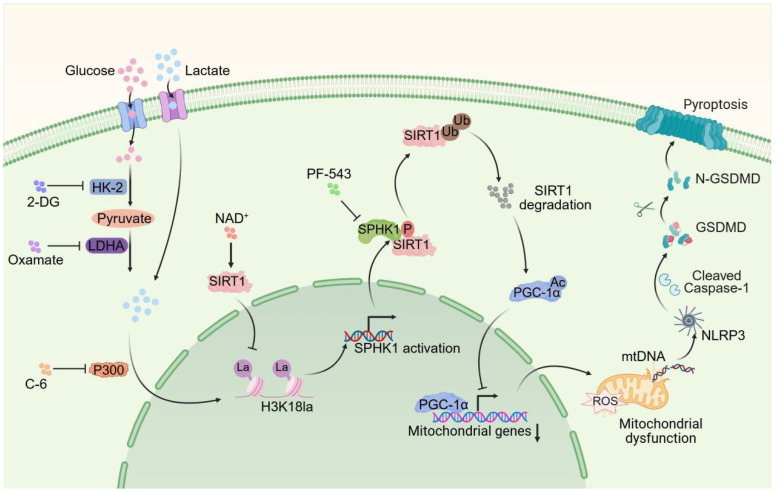
Schematic illustration of lactate exacerbating SA-AKI through H3K18la-mediated SPHK1-SIRT1 feedback loop accelerates pyroptosis in RTEC.

## Data Availability

All data generated are presented in the manuscript or supplementary files. Publicly available datasets analyzed were sourced from MIMIC-IV database (https://mimic.mit.edu/), GeneCards database (https://www.genecards.org/), GEO database (https://www.ncbi.nlm.nih.gov/geo/) and CPLM database (https://cplm.biocuckoo.cn/). Further datasets are available from the corresponding author upon reasonable request.

## Abbreviations

SA-AKI: sepsis-associated acute kidney injury
RTEC: renal tubular epithelial cells
LPS: lipopolysaccharide
CLP: cecal ligation and puncture
H3K18la: histone H3 lysine 18 lactylation
NLRP3: NOD-like receptor family pyrin domain containing 3
SPHK1: sphingosine kinase 1
SIRT1: sirtuin 1
PGC-1α: peroxisome proliferator-activated receptor gamma co-activator 1alpha
NAD^+^: nicotinamide adenine dinucleotide
KIM-1: kidney injury molecule-1
2-DG: 2-deoxy-D-glucose
OXA: oxamate
Fer-1: ferrostatin-1
3-MA: 3-methyladenine
Scr: serum creatinine
BUN: blood urea nitrogen
LDH: lactate dehydrogenase
MIMIC-IV: medical information mart for intensive care IV
DEGs: differentially expressed genes
Nig: nigericin
DAMP: damage-associated molecular pattern
mtDNA: mitochondrial DNA
TEM: transmission electron microscopy
mtROS: mitochondrial reactive oxygen species
ΔΨm: mitochondrial membrane potential
TOMM20: translocase of outer mitochondrial membrane 20
TFAM: mitochondrial transcription factor A
CHX: cycloheximide
Baf A1: Bafilomycin A1
co-IP: co-immunoprecipitation
CPLM: compendium of protein lysine modifications
ChIP: chromatin-immunoprecipitation
FDA: food and drug administration
PTMs: post-translational modifications
NAM: nicotinamide
NAMPT: nicotinamide phosphoribosyltransferase
GEO: gene expression omnibus
qRT-qPCR: quantitative real-time PCR
IHC: immunohistochemistry
GSEA: gene set enrichment analysis
