# Supplementary material for: H3K18 lactylation-mediated SPHK1-SIRT1 feedback loop accelerates pyroptosis of tubular epithelial cells in sepsis-associated acute kidney injury
Source: Theranostics. 2026 Feb 18;16(9):4768–86. doi: 10.7150/thno.122991 (PMC12964219; doi:10.7150/thno.122991)
Supplement: Supplementary file 1 — Supplementary figures and tables. [file thnov16p4768s1.pdf]

Supplementary material

H3K18 lactylation-mediated SPHK1-SIRT1 feedback loop accelerates pyroptosis of tubular epithelial cells in sepsis-associated acute kidney injury

*Yan Huang[1, #], Eryang Zhao[1, #], Guangyu Zhao[1, #], Wenfeng Zhuo[1], Yingsong Zhao[1],Hongda Wang[1], Guozheng Lv[1], Rong Hu[1], Zhu Zeng[1], Shengbo Han[1], Yuhang Hu[1] and Gang Zhao[1, *]*

Supplementary Figures:

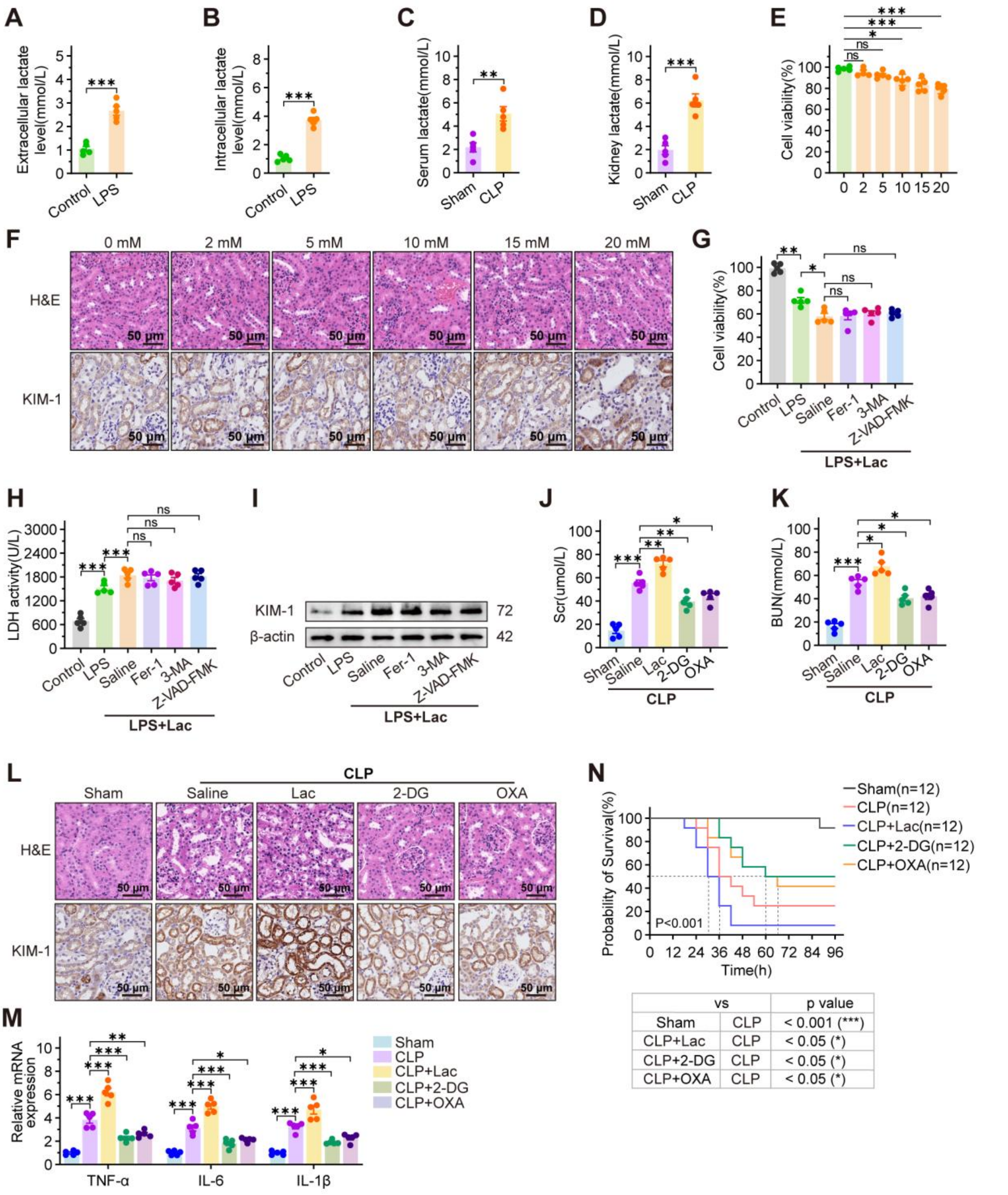

Figure S1. Hyperlactatemia aggravates the injury and inflammation of SA-AKI and was associated with poor prognosis. (A and B) Extracellular and intracellular lactate concentrations in HK-2 cells with or without LPS treatment (n = 5). (C and D) Serum and kidney lactate levels in mice with or without CLP induction (n = 5). (E) Cell viability in HK-2 cells treated with lactate at varying concentrations (n = 5). (F) Representative H&E staining and KIM-1 IHC images of kidney tissues from mice treated with lactate at varying concentrations (scale bar = 50 μm). (G) Cell viability,

(H) LDH release, (I) KIM-1 protein levels in HK-2[LPS] cells treated with lactate or lactate + Fer-1/3-MA/Z-VAD-FMK (n = 5). (J) Scr, (K) BUN, (L) representative H&E staining and KIM-1 IHC images of kidney tissue (scale bar = 50 μm), (M) kidney mRNA levels of inflammatory cytokines, and (N) survival rates in CLP mice treated with lactate/2-DG/oxamate (n = 5 for J–M; n = 12 for N). Data are mean ± SEM. *p < 0.05, **p < 0.01, and ***p < 0.001; ns, not significant.

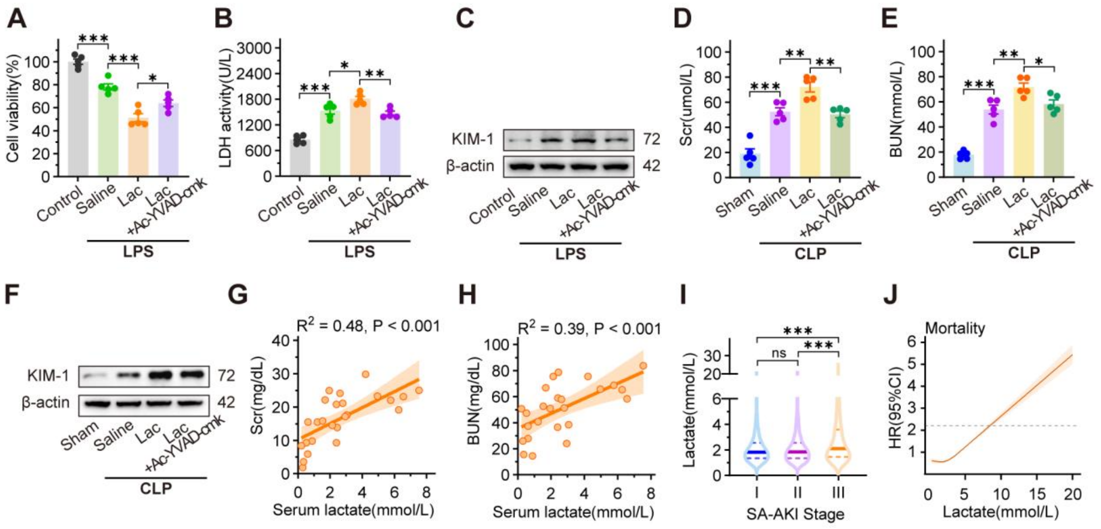

Figure S2. Lactate exacerbates SA-AKI via pyroptosis-dependent tubular damage. (A) Cell viability, (B) LDH release, and (C) KIM-1 protein expression in HK-2[LPS] treated with lactate or lactate + Ac-YVAD-cmk(n = 5). (D) Scr, (E) BUN, and (F) kidney KIM-1 protein levels in CLP mice treated with lactate or lactate + Ac-YVAD-cmk (n = 5). (G) Scr, and (H) BUN levels positively correlated with plasma lactate concentrations in SA-AKI patients (n = 25). (I and J) Elevated lactate predicted advanced AKI staging and increased mortality in SA-AKI patients (from MIMIC-III database analysis). Data are mean ± SEM. *p < 0.05, **p < 0.01, and ***p < 0.001; ns, not significant.

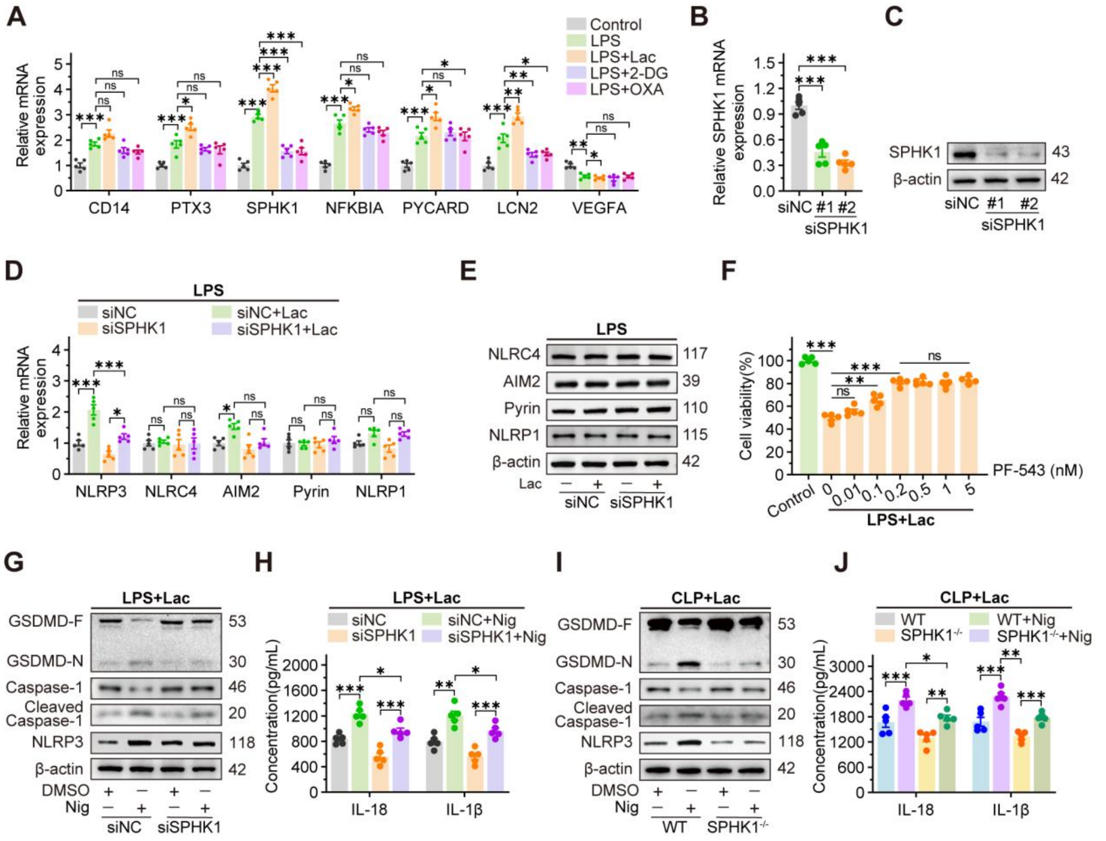

Figure S3. SPHK1 drives NLRP3-dependent pyroptosis by promoting mitochondrial DNA leakage. (A) mRNA expression levels of CD14, PTX3, SPHK1, NFKBIA, PYCARD, LCN2, and VEGFA in HK-2^{LPS} cells treated with lactate/2-DG/oxamate (n = 5). (B and C) SPHK1 mRNA and protein expression in HK-2 cells transfected with siNC or siSPHK1 (n = 5). (D and E) mRNA and levels of inflammasomes (NLRP3, NLRC4, AIM2, Pyrin, NLRP1) in HK-2^{LPS} cells transfected with siNC or siSPHK1, with or without lactate treatment (n = 5). (F) Cell viability of HK-2 cells treated with various PF-543 concentrations under LPS and lactate stimulation, compared to control (n = 5). (G) Protein expression of GSDMD, Caspase-1, Cleaved Caspase-1, NLRP3 and (H) secreted IL-18/IL-1β levels in lactated-treated HK-2^{LPS} cells transfected with siNC or siSPHK1, with or without nigericin pretreatment (n = 5). (I) Kidney protein expression of GSDMD, Caspase-1, Cleaved Caspase-1, and NLRP3 and (J) tissue IL-18/IL-1β levels in WT or SPHK1$^{-/-}$ CLP mice subjected to lactate, with or without nigericin pretreatment (n = 5). Data are mean ± SEM. *p < 0.05, **p < 0.01, and ***p < 0.001; ns, not significant.

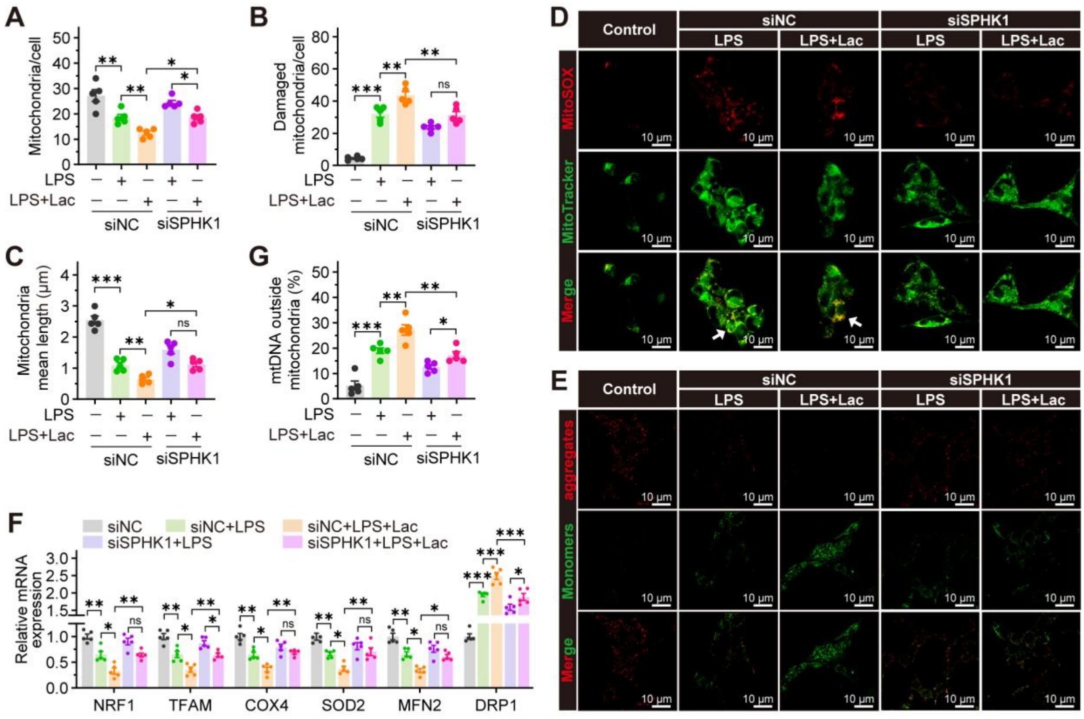

Figure S4. SPHK1 disrupts mitochondrial homeostasis by impairing the SIRT1-PGC-1α axis through phosphorylation-dependent inactivation. (A - C) Quantitative analysis of mitochondrial morphology in HK-2 cells by TEM (n = 5). (A) Mitochondrial number per cell, (B) damaged mitochondria count per cell, and (C) mitochondrial mean length. (D) Representative MitoSOX™ (Scale bars =10 μm) and (E) JC-1 fluorescence images (Scale bars =10 μm) in HK-2^{LPS} cells transfected with siNC or siSPHK1, with or without lactate treatment. (F) mRNA levels of mitochondrial-related genes and (G) extramitochondrial mtDNA levels based on Figure 3B in HK-2^{LPS} cells transfected with siNC or siSPHK1, with or without lactate treatment (n = 5). Data are mean ± SEM. *p < 0.05, **p < 0.01, and ***p < 0.001; ns, not significant.

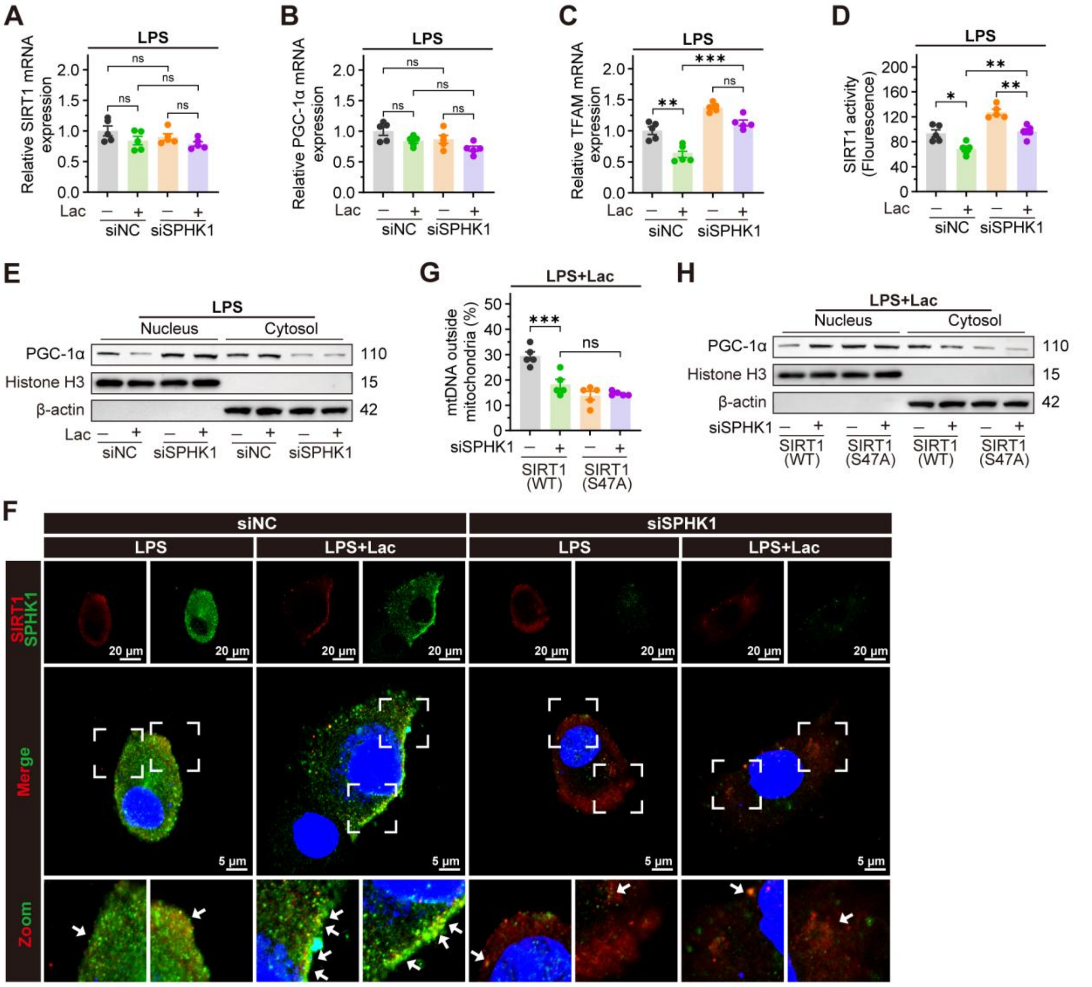

Figure S5. SPHK1 disrupts mitochondrial homeostasis by inducing ubiquitin-dependent SIRT1 degradation via phosphorylation at Ser47. (A - C) mRNA and levels of SIRT1, PGC-1α, and TFAM in HK-2^{LPS} cells transfected with siNC or siSPHK1, with or without lactate treatment (n = 5). (D) SIRT1 deacetylase activity and (E) subcellular localization of PGC-1α (nuclear vs. cytoplasmic) in HK-2^{LPS} cells transfected with siNC or siSPHK1, with or without lactate treatment (n = 5). (F) Representative immunofluorescence images of SPHK1 and SIRT1 in HK-2^{LPS} cells transfected with siNC or siSPHK1, with or without lactate treatment (Scale bars =20 μm, up; 4 μm, down). (G) Quantification of mtDNA leakage based on Figure 4J (n = 5). (H) Subcellular localization of PGC-1α in HK-2^{LPS} cells co-transfected with siNC or siSPHK1 together with wild-type SIRT1 (SIRT1-WT) or mutant SIRT1 (SIRT1-S47A) (n = 5). Data are mean ± SEM. *p < 0.05, **p < 0.01, and ***p < 0.001; ns, not significant.

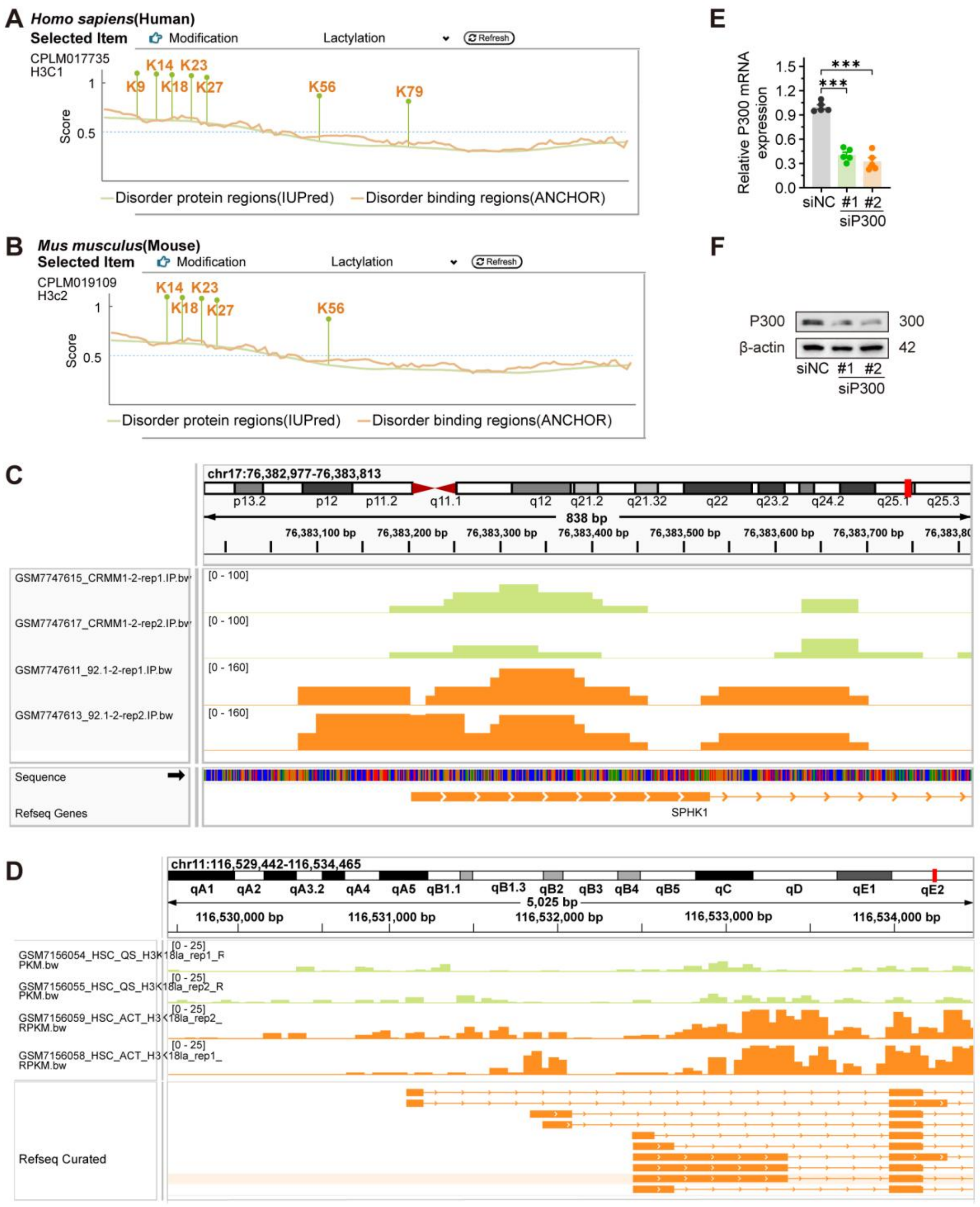

Figure S6. GEO database predicts H3K18la enrichment at the SPHK1 promoter region. (A and B) Comparative analysis of conserved histone lactylation sites in (A) human and (B) mouse histones using the CPLM database. (C and D) GEO database (GSE242018 and GSE229154) mining revealed H3K18la enrichment at the SPHK1 promoter region. (E and F) Validation of P300 knockdown efficiency by (E) qRT-PCR and (F) Western blot in HK-2 cells transfected with siNC or siP300 (n = 5). Data are mean ± SEM. *p < 0.05, **p < 0.01, and ***p < 0.001; ns, not significant.

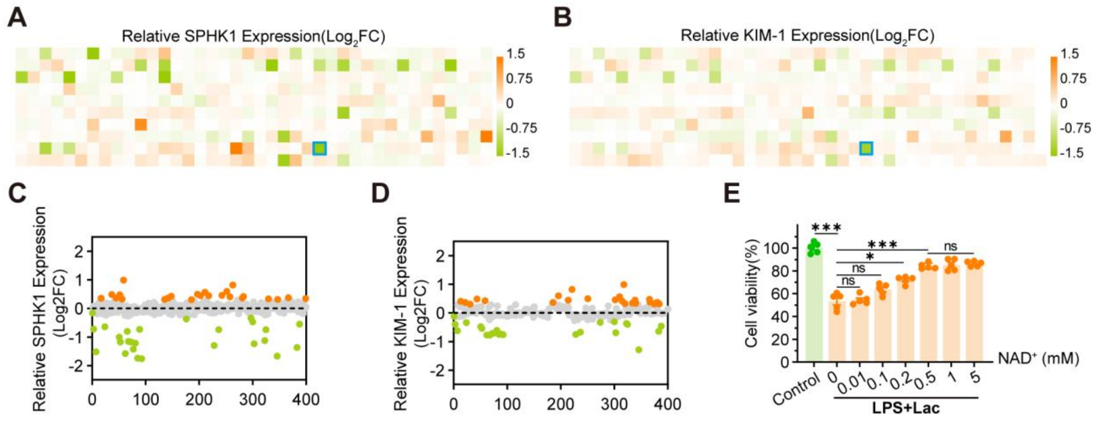

Figure S7. High-content imaging identifies 22 agents that coordinately downregulated both SPHK1 and KIM-1 expression. (A, B) Heatmaps and (C, D) scatter plots demonstrating drug efficacy in suppressing SPHK1 and KIM-1 expression. (E) Cell viability of HK-2 cells treated with various NAD^+ concentrations under LPS and lactate stimulation, compared to control (n = 5). Data are mean ± SEM. *$p < 0.05$, **$p < 0.01$, and ***$p < 0.001$; ns, not significant.

Supplementary tables

Table S1. Clinical Characteristics of the patients

Data of age, Scr, BUN, and lactate displayed as mean ± SEM (standard error of mean). Data of gender, hypertension, diabetes, cerebrovascular disease and pulmonary disease were presented as the occupation %. Scr: serum creatinine; BUN: blood urea nitrogen.

Characteristic	Patients (N=25)
Age, years	59±2
Gender (male)	14 (56%)
Scr	16.54±1.47
BUN	50.78±3.94
Lactate	2.64±0.42
Hypertension (%)	2 (8%)
Diabete (%)	3 (12%)
Cerebrovascular disease (%)	2 (8%)
Pulmonary disease (%)	4 (16%)

Table S2. The characteristics of included patients from MIMIC-IV database.

Clinical outcomes	Overall (n=17740)	< 2 (n=9464)	2≤; < 4 (n=6124)	4≤; < 10 (n=1883)	≥10 (n=269)
AKI (%)	17740 (100)	9464 (100)	6124 (100)	1883 (100)	269 (100)
Stage 1	4031 (22.7)	2292 (24.2)	1399 (22.8)	292 (4.8)	48 (17.8)
Stage 2	8206 (46.3)	4636 (49.0)	2914 (47.6)	617 (10.0)	39 (14.5)
Stage 3	5503 (31.0)	2536 (26.8)	1811 (29.6)	974 (15.9)	182 (67.6)
Discharged (%)	9781 (55.1)	5694 (60.1)	3408 (55.6)	657 (10.7)	22 (8.1)
Mortality (%)	8400 (47.3)	4211 (44.5)	2716 (44.3)	1226 (20.0)	247 (91.8)

Table S3. Sequence of siRNAs

Gene	Sequence	
Human SPHK1 siRNA-1	Sense (5' - 3')	GCAGCUUCCUUGAACCAUUTT
	Antisense (5' - 3')	AAUGGUUCAAGGAAGCUGCTT
Human SPHK1 siRNA-2	Sense (5' - 3')	GUGCACCCAAACUACUUCUTT
	Antisense (5' - 3')	AGAAGUAGUUUGGGUGCACTT
Human P300 siRNA-1	Sense (5' - 3')	CGACUUACCAGAUGAAUUAAU
	Antisense (5' - 3')	UAAUUCAUCUGGUAAGUCGUG
Human P300 siRNA-2	Sense (5' - 3')	GGAUUAGGUUUGAUAAAUAGC
	Antisense (5' - 3')	UAUUUAUCAAACCUAAUCCAG

Table S4. Primers Used for qRT-PCR Analysis.

Gene	Forward (5' - 3')	Reverse (5' - 3')
Human KIM-1	TGGCAGATTCTGTAGCTGGTT	AGAGAACATGAGCCTCTATTCCA
Human SPHK1	AGAGTGGGTTCCAAGACACCT	GGGTGCAGCAAACATCTCAC
Human CD14	ACGCCAGAACCTTGTGAGC	GCATGGATCTCCACCTCTACTG
Human PTX3	CATCTCCTTGCGATTCTGTTTTG	CCATTCCGAGTGCTCCTGA
Human NFKBIA	ACCTGGTGTCACTCCTGTTGA	CTGCTGCTGTATCCGGGTG
Human PYCARD	TGGATGCTCTGTACGGGAAG	CCAGGCTGGTGTGAAACTGAA
Human LCN2	GAAGTGTGACTACTGGATCAGGA	ACCACTCGGACGAGGTAACT
Human	AGGGCAGAATCATCACGAAG	AGGGTCTCGATTGGATGGCA

VEGFA	T	
Human NLRP3	GATCTTCGCTGCGATCAACAG	CGTGCATTATCTGAACCCCAC
Human NLRC4	TCAGAAGGAGACTTGGACGA T	GGAGGCCATTCAGGGTCAG
Human AIM2	TGGCAAAACGTCTTCAGGAG G	AGCTTGACTTAGTGGCTTTGG
Human NLPR1	GCAGTGCTAATGCCCTGGAT	GAGCTTGGTAGAGGAGTGAG G
Human SIRT1	TAGCCTTGTCAGATAAGGAAG GA	ACAGCTTCACAGTCAACTTTG T
Human PGC-1α	TCTGAGTCTGTATGGAGTGAC AT	CCAAGTCGTTCACATCTAGTT CA
Human NRF1	AGGAACACGGAGTGACCCAA	TATGCTCGGTGTAAGTAGCCA
Human TFAM	ATGGCGTTTCTCCGAAGCAT	TCCGCCCTATAAGCATCTTGA
Human COX4	CAGGGTATTTAGCCTAGTTGG C	GCCGATCCATATAAGCTGGG A
Human SOD2	GCTCCGGTTTTGGGGTATCTG	GCGTTGATGTGAGGTTCCAG
Human MFN2	CTCTCGATGCAACTCTATCGT C	TCCTGTACGTGTCTTCAAGGA A
Human DRP1	AAGGAGCCAGTCAAATTATTG	AGTCAACAAAGTCTCAGTATT
Human P300	TTCCCCTAACCTCAATATGGG AG	GCCTGTGTCATTGGGCTTTTG
Human β-actin	CATGTACGTTGCTATCCAGGC	CTCCTTAATGTCACGCACGAT
Mouse KIM-1	TGCACGCCATAATCAACCCTG	CATGCACTCACTTTTGCAGTT T
Mouse SPHK1	CCTAGTAGCTCCAAGTCAGAC A	ACCTTAGGTGGTCTTTGAGTC TC
Mouse TNF-α	TGGCCTCCCCTAACAGGAATA	GCCGAAAGCATTCTTAGTAGT GA
Mouse IL-6	GTCCCAGACATCAGGGAGTA A	TCGGATACTTCAGCGTCAGGA
Mouse IL-1β	CAACAAGGGTCCATCCTACGG	ATCTGGGCGGCCTACATCA
Mouse β-actin	GGCTGTATTCCCCTCCATCG	CCAGTTGGTAACAATGCCATG T

Table S5. Primers for ChIP-PCR, related to Figure 5.

Gene	Forward (5’ - 3’)	Reverse (5’ - 3’)
Human SPHK1	TCCCCTCGTTCCTGTTTCTC	GCCCCGTTTCCCAACACTT

Table S6. List of chemicals in screening

Number	Name	Number	Name
S1039	Rapamycin (AY-22989)	S1680	Disulfiram (NSC 190940)
S1717	Fomepizole	S2250	(-)-Epigallocatechin Gallate
S2303	Gossypol Acetate	S2289	Daidzin
S3729	Iron sucrose	S2391	Quercetin (NSC 9221)
S5742	Deferoxamine mesylate (Ba 33112)	S2610	Lonidamine (AF-1890)
S1150	Paclitaxel (NSC 125973)	S6400	Glucosamine
S1743	Esomeprazole magnesium	S8205	Enasidenib (AG-221)
S2310	Honokiol (NSC 293100)	S8206	Ivosidenib (AG-120)
S3791	Succinic acid	S2075	Rosiglitazone (BRL-49653) HCl
S5779	Trimetazidine	S2542	Phenformin (NSC-756501) HCl
S1208	Doxorubicin (Adriamycin) HCl	S2556	Rosiglitazone (BRL 49653)
S1950	Metformin HCl	S3763	Cinnamaldehyde
S2376	Ammonium Glycyrrhizinate	S4530	i-Inositol
S3850	Glucosamine sulfate	S4711	Esculetin
S8101	CB-5083	S5284	Adenosine 5'-monophosphate monohydrate
S1233	2-Methoxyestradiol (2-MeOE2)	S7953	ETC-1002
S2055	Gimeracil	S9285	Fargesin
S2396	Salidroside	P1114	Dasiglucagon
S3930	Liquiritin	S1707	Eplerenone (CGP 30083)
S8558	Tofogliflozin(CSG 452)	S1324	Doxazosin Mesylate
S1247	Leflunomide (HWA486)	S1442	Voriconazole (UK-109496)
S2233	Esomeprazole sodium	S1712	Deferasirox (ICL-670)
S2487	Mycophenolic acid	S1739	Thiabendazole
S4169	Teriflunomide	S1759	Pitavastatin (NK-104) calcium
S8615	Sodium dichloroacetate (DCA)	S1792	Simvastatin (MK 733)
S1396	Resveratrol (SRT501)	S1794	Fenofibrate (NSC-281319)
S2295	Emodin	S1831	Carvedilol
S2528	Ciclopirox	S1937	Isoniazid

S4701	2-Deoxy-D-glucose (2-DG)	S1949	Menadione (NSC 4170)
S6626	Brequinar (DUP785)	S2046	Pioglitazone HCl
S1404	Trilostane	S2246	Abiraterone Acetate (CB7630)
S2296	Enoxolone	S2268	Baicalein
S3019	Ciclopirox ethanolamine	S2314	Kaempferol (NSC 407289)
S4723	(-)Epicatechin	S2329	Naringin
S6852	Gossypol	S2390	Polydatin
S1501	Mycophenolate mofetil (RS-61443)	S2606	Mifepristone (RU486)
S2302	Glycyrrhizin (NSC 167409)	S3017	Aspirin (NSC 27223)
S3694	Glucosamine hydrochloride	S3046	Azilsartan
S5454	Saikosaponin D	S3114	Vitamin C
S4013	Sodium Monofluorophosphate	S3612	Rosmarinic acid
S5097	Methotrexate disodium	S3868	Harmine
S5545	DL-Serine	S3957	Gamma-Oryzanol
S1007	Roxadustat (FG-4592)	S4155	Chlorzoxazone
S1500	Betamethasone	S4255	Quinacrine 2HCl
S1690	Betamethasone Valerate	S4539	Salicylic acid
S1987	Mometasone furoate	S4686	Vitamin E
S2570	Prednisolone Acetate	S4707	Oleic Acid
S3781	Ginkgolide C	S5082	Vitamin K2
S4752	Corticosterone (NSC-9705)	S6281	Sodium Thiocyanate
S5901	Canagliflozin hemihydrate	S7456	Osilodrostat (LCI699)
S8637	Ipragliflozin (ASP1941)	S9046	Berberine
S1033	Nilotinib (AMN-107)	S5922	L-Carnitine hydrochloride
S1548	Dapagliflozin (BMS-512148)	S9925	Imeglimin (EMD 387008) Hydrochloride
S1696	Hydrocortisone (NSC 10483)	S1857	Etidronate
S1992	Fluticasone propionate	S1037	Perifosine (KRX-0401)
S2584	Clobetasol propionate	S3056	Miltefosine
S3810	Scutellarin	S3753	L-Leucine
S5325	Nitisinone	S4430	Hydroxychloroquine Sulfate (NSC 4375)
S6010	Sodium L-lactate	S5526	β-Alanine
S9111	Isorhamnetin	S6266	(S)-Glutamic acid
S1044	Temsirolimus (CCI-779)	S2251	(-)-Huperzine A (HupA)
S1614	Riluzole (PK 26124)	S1911	Disodium Cromoglycate
S1701	Desonide	S1271	Acarbose
S2123	Dextrose	S2489	Nateglinide
S2608	Fluocinonide	S4204	Isosorbide
S4088	Flumethasone	S5083	Lentinan

S5360	Diflorasone	S5717	Acetohexamide
S6221	Methyl cinnamate	S9002	L-Fucose
S1120	Everolimus (RAD001)	S2416	Chondroitin sulfate
S1622	Prednisone (NSC-10023)	S2035	Epalrestat (ONO-2235)
S1733	Methylprednisolone (NSC-19987)	S1346	Heparin sodium
S2226	Idelalisib	S1312	Streptozotocin (STZ)
S2760	Canagliflozin (JNJ 28431754)	S2589	Miglitol
S4228	Fluorometholone Acetate	S4543	Trimetazidine dihydrochloride
S5413	Ertugliflozin	S5102	Stachyose
S6459	Nifurtimox	S5763	D-Mannose
S1245	Latrepirdine 2HCl	S9349	D-(+)-Raffinose pentahydrate
S1628	Triamcinolone Acetonide	S4174	Sodium Gluconate
S1737	Prednisolone (NSC-9900)	S4218	Amoxapine
S2347	Quercetin Dihydrate	S3749	Calcium gluconate monohydrate
S2809	MPEP	S1426	Repaglinide
S4268	Flufenamic acid	S2609	Inulin
S5486	Fluorometholone	S4546	Xylitol
S7028	Duvelisib (IPI-145)	S5134	D(-)-Arabinose
S1282	Artemisinin	S5830	L-Xylose
S1669	Loteprednol etabonate	S9192	Vitexin
S1854	Bifonazole	S4704	D-(+)-Cellobiose
S2357	Silibinin (NSC 651520)	S1511	Lactulose
S2814	Alpelisib (BYL719)	S3772	5-Hydroxymethylfurfural
S4299	Dicoumarol	S4610	Mebendazole
S5501	Hydrocortisone acetate	S5155	Raffinose
S7954	CP21R7 (CP21)	S6121	Maltotriose
S1286	Budesonide	S9270	Dracohodin perochlorate
S1688	Betamethasone Dipropionate	S2124	Xylose
S1888	Deflazacort	S3849	D-Galactose
S2470	Fluocinolone Acetonide	S4768	Melibiose
S3070	Piracetam	S5176	Fructose
S4561	Danthron	S6257	N-Acetylglucosamine
S5566	Dapagliflozin propanediol monohydrate	S3101	2-Deoxy-D-ribose
S8022	Empagliflozin (BI 10773)	S2258	Esculin
S1342	Genistein (NPI 031L)	S3887	α-L-Rhamnose monohydrate
S1689	Meprednisone	S4792	N-Acetylneuraminic acid
S1933	Triamcinolone	S5544	D-Ribose
S2559	Cortisone acetate	S6346	L-(+)-Arabinose
S3078	Beclomethasone	S2317	L-(+)-Rhamnose Monohydrate

	dipropionate		
S4716	Evans Blue	S3986	L-Arabinose
S5685	Desoximetasone	S4830	Maltose
S8103	Sotagliflozin (LX4211)	S5554	Lanatoside C
S1042	Sunitinib (SU11248) malate	S6443	Chromium picolinate
S1204	Melatonin (NSC 113928)	S2351	Salicin
S1389	Omeprazole	S4101	Voglibose
S1640	Albendazole (SKF-62979)	S4868	Xanthinol Nicotinate
S1905	Amlodipine	S5580	Arabic gum
S2271	Berberine chloride (NSC 646666)	S7852	Eliglustat
S2339	Paeonol	S3944	Valproic acid (VPA)
S2485	Mitoxantrone (NSC-301739) 2HCl	S4164	Doxofylline
S3212	Moclobemide (Ro 111163)	S4353	Terfenadine
S3783	Echinacoside	S4706	Eugenol
S1047	Vorinostat (SAHA)	S4862	Squalene
S1210	Methotrexate (CL-14377)	S5266	Stiripentol
S1472	Safinamide Mesylate	S5550	Ethyl gallate
S1667	Trichlormethiazide	S7536	Lorlatinib (PF-6463922)
S1972	Tamoxifen (ICI 46474) Citrate	S9024	3-O-Acetyl-11-keto-β-boswellic acid (AKBA)
S2280	Chlorogenic Acid	S3593	Rhodamine 6G
S2341	(-)-Parthenolide	S3969	Veratric acid
S2492	Novobiocin Sodium (NSC 2382)	S4166	Chlorpropamide
S3616	Asiaticoside	S4538	Pantoprazole sodium
S3817	Harmine hydrochloride	S4717	Isatin
S1060	Olaparib (AZD2281)	S4953	Usnic acid
S1225	Etoposide (VP-16)	S5357	Safinamide
S1547	Febuxostat	S5623	Bedaquiline
S1681	Mesalamine (5-ASA)	S7781	Sunitinib (SU11248)
S1990	Capsaicin(Vanilloid)	S9086	Dihydrocapsaicin
S2300	Ferulic Acid	S4419	Ensulizole
S2348	Rotenone (Barbasco)	S3998	(+)-α-Lipoic acid
S2493	Olanzapine	S4188	Sasapyrine
S3643	Amitraz	S4601	Clioquinol
S3824	Quercitrin	S4722	(+)-Catechin
S1068	Crizotinib (PF-02341066)	S4999	Avermectin B1
S1238	Tamoxifen (ICI 46474)	S5369	Ethoxyquin
S1619	Prilocaine	S5663	Minaprine dihydrochloride
S1693	Carbamazepine	S7787	Docetaxel Trihydrate
S1994	Lacidipine	S9519	Fadrozole (CGS16949A)

S2309	Hesperidin	S4425	Rifamycin S
S2350	Rutin	S4007	Pentamidine isethionate
S2553	5-Aminolevulinic acid HCl	S4206	Cysteamine HCl
S3654	Tauroursodeoxycholic Acid (TUDCA)	S4603	Gallic acid
S3842	Isoquercitrin	S4753	Ganoderic acid A
S1119	Cabozantinib (BMS-907351)	S5037	Phenazine methosulfate
S1322	Dexamethasone (MK-125)	S5372	Methyl Aminolevulinate Hydrochloride
S1623	Acetylcysteine (N-acetylcysteine)	S5703	Carvedilol Phosphate
S1716	Glyburide (Glibenclamide)	S7867	Oleuropein
S2062	Tiopronin	S3273	Hypericin
S2320	Luteolin	S4483	Iproniazid phosphate
S2358	Silymarin	S4017	Allylthiourea
S2554	Daphnetin	S4246	Tranylcypromine (2-PCPA) HCl
S3666	Ilaprazole	S4612	Dapson
S3883	Protopine	S4779	Menadiol Diacetate
S1148	Docetaxel (RP56976)	S5058	Revaprazan Hydrochloride
S1351	Ivermectin (MK-933)	S5435	Quinacrine Dihydrochloride Dihydrate
S1630	Allopurinol	S5709	Norgestrel
S1774	Thioguanine (NSC 752)	S8016	Vonoprazan Fumarate (TAK-438)
S2102	Rasagiline Mesylate	S3301	Cynarin
S2331	Neohesperidin Dihydrochalcone (Nhdc)	S4489	Tetraethylammonium chloride
S2369	Troxerutin	S4099	Dexlansoprazole
S2586	Dimethyl Fumarate	S4256	Buspirone HCl
S3690	Pargyline hydrochloride	S4630	Diazoxide
S3885	Pyrogallol	S4845	Rabeprazole
S1168	Valproic Acid (NSC 93819) sodium salt	S5159	Doxycycline
S1354	Lansoprazole	S5440	Berberine Sulfate
S1631	Allopurinol Sodium	S5795	Rasagiline
S1848	Curcumin	S8048	Venetoclax (ABT-199)
S2105	Pantoprazole	S3143	Pyruvic acid
S2332	Neohesperidin	S4531	Iohexol
S2370	Ursolic Acid	S4100	Esmolol HCl
S2605	Idebenone	S4263	Efaproxiral Sodium
S3722	Isavuconazole	S4662	Atazanavir
S3924	Ginsenoside Rb1	S4853	Ecabet sodium

S1185	Ritonavir (ABT-538)	S5208	Ciprofloxacin hydrochloride hydrate
S1378	Ruxolitinib (INCB018424)	S5452	Sanguinarine chloride
S1638	Ibuprofen (NSC 256857)	S6441	Riboflavin Tetrabutyrate
S1899	Nicotinamide (NSC 13128)	S9032	Sanguinarine
S2263	Arbutin	S6959	Perhexiline maleate
S2337	Osthole	S4929	Enasidenib Mesylate
S2423	(S)-10-Hydroxycamptothecin	S4104	Diminazene Aceturate
S3124	Dexamethasone Acetate	S4284	Chloroprocaine HCl
S3755	Betaine	S4698	Vitamin K1
S3925	(-)-Epicatechin gallate	S4856	Iproniazid
S5608	Pantoprazole sodium hydrate	S5243	Ruxolitinib Phosphate
S5958	Metformin	S5502	Ilaprazole sodium
S9783	6-Aminonicotinamide	S6462	Amezinium (methylsulfate)
E0165	Phenelzine sulfate	S9338	Octyl gallate
E0680	Deslanoside	S3577	Rhodamine 123
S1217	Cyclophosphamide	S5190	Crizotinib hydrochloride
S6594	Liarozole dihydrochloride	S4933	Lithium carbonate
S2518	NAD+	S3096	L-Histidine monohydrochloride monohydrate
S3852	L-Theanine	E0375	Ammonium ferric citrate
S3973	L-SelenoMethionine	S1046	Vandetanib (ZD6474)
S4298	Amifostine trihydrate	S1837	Flubendazole
S4606	Glutathione	S4033	Sennoside A
